# A Combined Chemoinformatics- and Machine Learning-Based Approach Identifies Chlormidazole as a Drug Repurposing Candidate against Aggressive Prostate Cancer

**DOI:** 10.1021/acs.jmedchem.5c03833

**Published:** 2026-08-04

**Authors:** Leonardo Bernal, Luca Pinzi, Tommaso Martinelli, Arianna Rinaldi, Isabella Piccinini, Silvia Belluti, Nicolò Bisi, Carol Imbriano, Giulio Rastelli

**Affiliations:** † Department of Life Sciences, 9306 University of Modena and Reggio Emilia, Via Giuseppe Campi 103, Modena, 41125, Italy; ‡ Clinical and Experimental Medicine PhD Program, University of Modena and Reggio Emilia, Via Giuseppe Campi 287, Modena 41125, Italy

## Abstract

Despite recent therapeutic advances, treatment options for advanced, therapy-resistant, and metastatic prostate cancer (PCa) remain limited. Here, we developed and prospectively validated an integrated chemoinformatics and machine learning (ML) workflow with ligand-based similarity filtering. Validation on independent external data sets showed that this applicability-domain-guided integration strategy can reduce false positives and improve virtual screening performance. Screening of DrugBank identified five repurposing candidates with confirmed antiproliferative activity in both 2D and 3D PCa models. Among them, the antifungal agent chlormidazole emerged as the most promising candidate, displaying tumor-selective and predominantly cytostatic activity associated with p57 upregulation, reduced Rb phosphorylation, and G1 arrest. Chlormidazole also enhanced the antiproliferative activity of docetaxel in both models, achieving comparable efficacy at substantially lower docetaxel concentrations. These findings identify chlormidazole as a promising repurposing candidate for PCa and demonstrate the value of integrating chemoinformatics with ML for drug repurposing and virtual screening.

## Graphical abstract

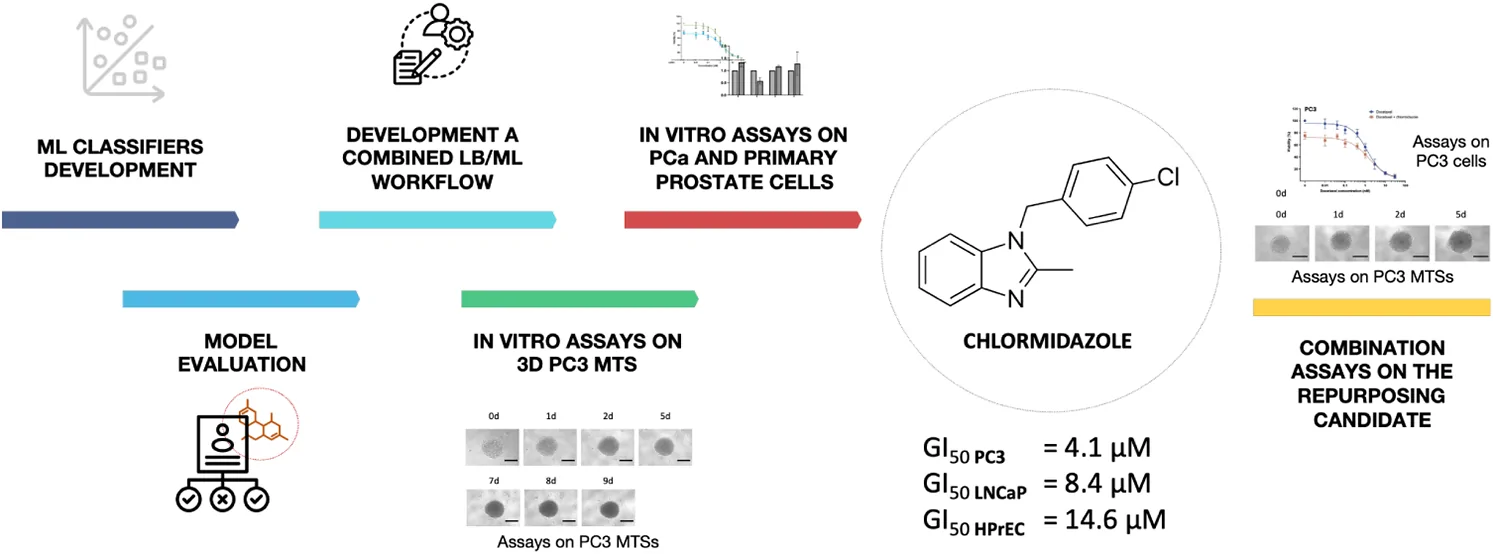

## Introduction

Prostate cancer (PCa) accounts for more than 350,000 deaths annually and represents the second most frequent malignancy among men worldwide. Its incidence continues to rise, particularly in Western countries.^1^ For patients diagnosed at early stages, several therapeutic options are available, including radiotherapy and radical prostatectomy, often combined with androgen deprivation therapy (ADT) or next-generation androgen receptor pathway inhibitors (ARPIs), as well as pelvic lymph node dissection for localized and high-risk cases.^2^ These interventions have substantially improved clinical outcomes, with five-year survival rates exceeding 80% for early-stage PCa.^3^ In contrast, advanced and aggressive forms of the disease, including androgen receptor (AR)-independent prostate cancer, previously referred to as castration-resistant prostate cancer (CRPC), and its metastatic forms (mPCa, mCRPC), remain associated with a markedly poorer prognosis. In patients with distant metastases, 5-year survival rates drop to approximately 30–40%.^3,4^

Although several drugs have recently received approval (e.g., abiraterone, enzalutamide, and apalutamide) and others are currently under clinical investigation (e.g., new poly-ADP ribose polymerase inhibitors),^5^ their efficacy against CRPC and mCRPC remains limited. Consequently, there is a continuing need for more effective therapeutic strategies, particularly for advanced-stage PCa.

Drug repurposing has emerged as an attractive alternative or complement to *de novo* drug discovery, as it allows circumventing challenges associated with early-stage discovery and development, including toxicity and safety issues, high costs, and lengthy timelines.^6,7^ By leveraging “de-risked”, clinically characterized compounds, drug repurposing can substantially reduce development timelines and attrition risks. This strategy can be supported by information available in public repositories, such as DrugBank^8^ and ChEMBL,^9^ as well as by computational approaches such as chemoinformatics and machine learning (ML).^10^ Among computational methodologies, ligand-based similarity approaches have historically played a central role in drug discovery. These methods have proven effective for identifying drug repurposing opportunities across a broad range of therapeutic areas, including cancer and infectious diseases.^11,12,13^ Their strengths and limitations have been extensively reviewed.^14,15,16,17^ Nevertheless, several intrinsic limitations remain.^17^ Structurally similar compounds may display markedly different biological activities, a phenomenon commonly referred to as “activity cliffs”.^18^ Moreover, structural similarity does not necessarily correlate with potency,^19^ and commonly used similarity metrics, such as the Tanimoto coefficient, may introduce intrinsic biases that compromise predictive reliability.^20,21,22^

In parallel, ML-based methods have gained increasing importance in drug discovery.^16,23^ These approaches enable the development of classifiers and quantitative structure–activity relationship (QSAR) models capable of identifying biologically active compounds within large chemical data sets.^23,24,25^ However, predictive performance is strongly dependent on the quality, size, and representativeness of the training data.^26^ As a consequence, ML models typically perform best when applied to compounds falling within or near the chemical space represented during training.^27,28,29^ Furthermore, unlike traditional ligand-based (LB) and structure-based (SB) methods, standard ML classifiers do not inherently provide compound-ranking scores, which limits their utility for candidate prioritization in virtual screening campaigns.

To the best of our knowledge, previously reported LB/ML workflows generally apply similarity methods and machine learning either independently or as parallel prioritization strategies, while relatively few studies have investigated how these methodologies can be integrated to address applicability-domain limitations and improve prediction reliability on structurally diverse compounds.^30,31,32^ On these premises, in this study, we developed a computational strategy that integrates chemoinformatics and ML methodologies (hereafter referred to as the *Combined approach*). In particular, in this framework, ligand-based similarity filtering is employed as a chemical space-focusing step prior to ML classification. By prioritizing compounds located in regions of chemical space enriched in known active molecules, this approach is intended to improve compliance with the applicability domain of the ML models and reduce extrapolation toward structurally distant chemotypes. The resulting subset of prioritized compounds is subsequently evaluated by using ML classifiers to further refine candidate selection, thereby combining the complementary strengths of both methodologies. This strategy was specifically designed to improve prediction reliability when screening structurally diverse chemical spaces and was systematically evaluated using fully independent external data sets.

Building on our previous work,^33^ we applied the *Combinedapproach* to identify drug repurposing candidates against PCa. The most promising compounds were evaluated *in vitro* using both 3D spheroid models derived from PC3 PCa cells and 2D cultures of androgen-independent PC3 and androgen-responsive LNCaP cells. Tumor selectivity was also assessed using primary prostate epithelial cells (HPrEC), leading to the identification of chlormidazole as a particularly promising candidate because of its marked antiproliferative activity against PCa cells relative to nonmalignant prostate cells. To further investigate its biological activity, cell cycle analyses and mechanistic studies were performed to determine whether chlormidazole exerts a predominantly cytostatic or cytotoxic effect. Finally, chlormidazole was evaluated in combination with docetaxel in both 2D and 3D PCa models to assess its potential in combination therapy strategies.

This study reports the methodological implementation and validation of our integrated computational framework, together with the prospective experimental evaluation of repurposed drug candidates. Beyond identifying novel candidates against PCa, a primary objective of this work was to evaluate whether the applicability-domain-guided integration of LB-similarity and ML approaches could improve virtual screening performance under realistic external validation conditions. The proposed strategy is customizable and broadly applicable to large-scale virtual screening and drug-repurposing campaigns.

## Results and Discussion

Here, we developed and prospectively validated a workflow that integrates ligand-based similarity analysis with machine-learning classification models to prioritize drug repurposing candidates against prostate cancer. In our previous study,^33^ we identified a subset of DrugBank compounds with 2D similarity scores above the defined threshold with respect to ChEMBL ligands with known antiproliferative activity against androgen-responsive and androgen-nonresponsive PCa cell lines. Notably, these drugs had not previously been tested against PCa, which makes them an excellent set of candidates for further evaluation. Building on those findings, the present study aimed to assess whether the applicability-domain-guided integration of LB similarity filtering and ML classification could improve virtual screening performance while facilitating the identification of biologically relevant repurposing candidates. The overall computational and experimental workflows are summarized in Figure 1 and described below.

**Figure 1:**
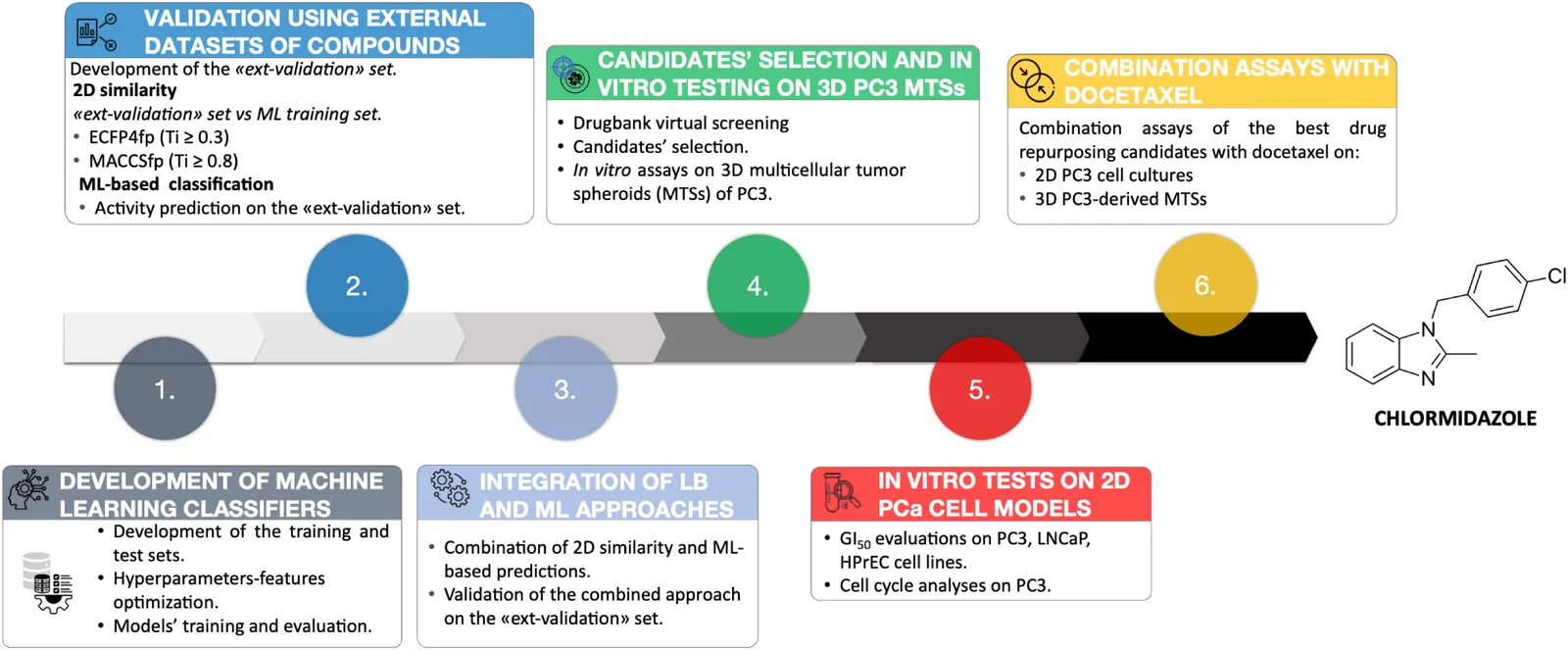
Drug repurposing workflow adopted in this study.

### Development of Machine-Learning Classifiers to Predict Antiproliferative Activity in Prostate Cancer Cell Lines

Machine-learning classifiers trained on ChEMBL ligands were developed to predict the antiproliferative activity of compounds against selected PCa cell lines. Data sets of compounds with reported antiproliferative activity against PC3, DU145, and LNCaP cells were retrieved from ChEMBL29 for consistency with our previous studies.^33,34^ After curation and filtering (see Experimental section), the resulting data set contained 10505, 9004, and 3002 compounds, respectively. Compounds were classified as “active” or “inactive” using a 10 μM activity threshold (Figure S1), a criterion widely reported in the literature and adopted in high-throughput screening programs,^35^ including the National Cancer Institute NCI-60 human cancer cell line screening platform. In addition to providing a standardized framework for activity classification, this threshold resulted in reasonably balanced data sets suitable for classifier development (Figure S1). Analysis of data set composition revealed substantial overlap among the three cell lines, with (i) 689 compounds tested against all three cell lines; (ii) 2492 tested against PC3 and DU145; (iii) 1118 tested against PC3 and LNCaP, and (iv) 1265 tested against DU145 and LNCaP (Figure S2). Notably, the PC3 and DU145 data sets were substantially larger than that of LNCaP. Following curation, 220 RDKit^36^ molecular descriptors encompassing a wide range of molecular properties were calculated for each compound. Descriptors containing missing or non-numeric values were removed. Then, each data set was clustered and split into training and test sets (70:30 ratio) using a custom strategy designed to preserve class balance while maintaining chemical diversity and addressing model overfitting/underfitting (Table 1 and Figure S3). All model-dependent preprocessing steps were performed exclusively on the training data and subsequently applied to the test set. Molecular descriptors with near-zero variance or high pairwise correlation were removed, and the remaining features were standardized using the RobustScaler implementation available in Scikit-Learn to standardize feature values and minimize the influence of outliers.^37^

**Table 1:** Size and Composition of the Training and Test Sets, and Features’ Selection Results for Each PCa Cell Line

Data set	Number of compounds in the training set	Number of compounds in the test set	% Active compounds in the training set	% Active compounds in the test set	Number of features retained after ML models development
**PC3**	6628	3877	60%	58%	108
**DU145**	5745	3258	56%	56%	196
**LNCaP**	1934	1067	67%	66%	50

To ensure a balanced representation of “active” and “inactive” compounds, StratifiedKFold cross-validation was applied in both feature selection and hyperparameter optimization, thereby maintaining similar class distributions across all folds. During each iteration, one fold was used for validation, and the remaining k–1 folds were used for training. Model development was conducted using 10-fold stratified cross-validation implemented through the GridSearchCV function of Scikit-Learn (Table S1). Hyperparameters were initially optimized using the complete set of molecular descriptors. Feature selection was subsequently performed using Recursive Feature Elimination with Cross-Validation (RFECV)^38^ combined with Matthews Correlation Coefficient (MCC)^37^ as the optimization metric. MCC was chosen for its robustness in binary classification tasks and its ability to handle imbalanced classes.^39^ During RFECV, descriptors were iteratively removed according to their contribution to predictive performance until the optimal subset of features was identified, thereby reducing model complexity while improving generalization. A second round of hyperparameter fine-tuning was then performed on the reduced set of descriptors to account for potential changes in model behavior resulting from feature elimination. The final optimized models retained 108, 196, and 50 descriptors for the PC3, DU145, and LNCaP data sets, respectively (Table 1 and S2).

Classifiers were built using the Extra Trees algorithm implemented with the Scikit-Learn python library.^40^ In a recent benchmarking study performed on the same data sets, Random Forest (RF), Extra Trees (ET), Gradient Boosting Machines (GBM), and XGBoost (XGB) models showed overall comparable predictive performance, with GBM and XGB achieving slightly higher MCC and F1-score values in specific cases (Table S3).^33^ Nevertheless, ET was selected for the present study because ensemble tree-based methods are particularly well suited for cheminformatics applications involving high-dimensional and heterogeneous molecular descriptor spaces,^41^ such as those generated by RDKit.^36^ In addition to providing robust predictive performance, ET offers several practical advantages, including computational efficiency, reduced susceptibility to overfitting, stable predictive performance, and robustness toward structurally diverse and potentially noisy data sets.^42^ Unlike boosting-based approaches, ET introduces additional randomization during tree construction, which generally improves generalization and reduces sensitivity to correlated descriptors and noisy activity annotations frequently observed in ChEMBL-derived data sets.

MCC was selected as the primary evaluation metric. Because the curated data sets showed approximately balanced distributions of active and inactive compounds (Figure S1), MCC provided a comprehensive assessment of model quality by simultaneously accounting for true positives (TP), false positives (FP), true negatives (TN), and false negatives (FN).

The developed classifiers demonstrated consistently good performance across all evaluation metrics in both the training and test sets (Table 2 and Figure S4). Notably, MCC values of approximately 0.6 were obtained on the independent test sets, indicating a robust ability to discriminate between active and inactive compounds. High accuracy, precision, F1-score, and ROC AUC values further confirmed the predictive ability of the models across the three cell lines. The adopted training/test set split strategy ensured that the models were not biased toward specific chemical scaffolds, thereby enhancing their ability to generalize to previously unseen compounds. In addition, the combined use of stratified K-fold cross-validation, RFECV feature selection, and hyperparameter optimization contributed to reducing overfitting while improving model robustness and generalization.

**Table 2:** Performance Metrics of Each Developed ML Classifier for Both Training and Test Sets of PC3, DU145, and LNCaP Cell Lines

	PC3		DU145		LNCaP	
Metric	Training set	Test set	Training set	Test set	Training set	Test set
MCC	0.79	0.59	0.83	0.60	0.77	0.59
Accuracy	0.89	0.79	0.91	0.80	0.89	0.81
Precision	0.97	0.86	0.91	0.81	0.97	0.88
Recall	0.85	0.76	0.94	0.86	0.86	0.82
F1 Score	0.90	0.81	0.93	0.83	0.91	0.85
ROC AUC	0.97	0.88	0.97	0.88	0.97	0.90

Collectively, these results indicate that the developed classifiers were able to effectively identify relevant structure–activity relationships within the chemical space represented in the training data sets and maintain satisfactory predictive performance on independent test sets.

### Validation Using External Data Sets of Compounds

To validate the developed workflow, we used fully independent external validation data sets derived from ChEMBL34, hereafter referred to as *ext-validation* sets. These sets comprised compounds with documented antiproliferative activity against PC3, DU145, and LNCaP cells that were absent from the ChEMBL29 data set used for model development. After curation and filtering, the *ext-validation* sets contained 2292, 1054, and 464 compounds for PC3, DU145, and LNCaP, respectively.

To establish a robust benchmark for the proposed workflow, LB similarity screening and ML classification were initially evaluated independently. Subsequently, the two methodologies were integrated, and their performance was compared under identical validation conditions.

For validation of the similarity-based approach, compounds in the *ext-validation* sets were compared against the ChEMBL29 compounds used as queries. This allowed us to (i) evaluate the ability of ligand-based similarity screenings to correctly rank active (TP) versus inactive (FP) compounds; (ii) ensure consistency with results reported in our previous study;^33^ and (iii) evaluate the advantage of combining ligand-based and ML approaches in a virtual screening context (as detailed in the following paragraph). The *Precision* parameter was used to assess the ability of ligand-based similarity screenings to identify TP compounds within ranked lists and to compare this performance with that of ML classifiers. In this context, high precision indicates strong enrichment of genuinely active compounds among the top-ranked candidates. Because LB approaches generate ranked lists rather than binary classifications (i.e., “active” or “inactive”), *Precision* was selected as the primary metric for evaluating screening performance. The similarity-based approach successfully enriched active compounds across all three data sets (Table 3 and Supporting File 1). In the PC3 validation set, the LB method retrieved 29% of the compounds (i.e., 685 molecules), 79% of which were true actives, corresponding to 23% of all known active compounds in the validation set. Similar results were obtained for DU145, where 28% of the compounds (i.e., 308 molecules) were retrieved with a true positive rate of 72%, representing 21% of all true actives in the *ext-validation* set. In contrast, lower performance was observed for LNCaP, where only 19% of the compounds (i.e., 88 molecules) were retrieved, 48% of which were true actives, corresponding to only 9% of total actives. *Precision* values reflected these trends, being high for PC3 (0.79) and DU145 (0.72), but lower for LNCaP (0.48) (Table 3).

**Table 3:** Results of the Ligand-Based Similarity Estimations Performed on the External Validation Sets for Each PCa Cell Line

	PC3		DU145		LNCaP	
	Selected according to similarity thresholds	Not selected according to similarity thresholds	Selected according to similarity thresholds	Not selected according to similarity thresholds	Selected according to similarity thresholds	Not selected according to similarity thresholds
Number of molecules	685 (536)^a^	1698 (870)^a^	308 (224)^a^	773 (382)^a^	88 (42)^a^	380 (278)^a^
Precision	0.79		0.72		0.48	

a Number of true active compounds.

These results indicate that similarity-based screening effectively enriches active compounds but still generates a substantial number of false positives, particularly in chemically heterogeneous data sets.

The developed ML classifiers were subsequently evaluated using the same *ext-validation* data sets (Supporting Information 2). As reported in Table 4, all classifiers retained meaningful predictive capability despite the substantially more challenging validation conditions. The LNCaP classifier displayed the strongest performance, achieving *precision, recall*, F1-score, and ROC-AUC of 0.80, 0.83, 0.82, and 0.69, respectively.

**Table 4:** Results of the Developed Machine Learning Classifiers Applied to the *Ext-Validation* Sets for Each PCa Cell Line^a^

	PC3		DU145		LNCaP	
	Actives	Inactives	Actives	Inactives	Actives	Inactives
Number of predicted molecules	1355 (955)^b^	1016 (451)^b^	749 (509)^b^	332 (97)^b^	335 (267)^b^	133 (53)^b^
Percentages of molecules	TP: 41,7%	TN: 22.9%	TP: 48.3%	TN: 22.0%	TP: 57.5%	TN: 16.8%
	FP: 15.7%	FN: 19.7%	FP: 20.5%	FN: 9.2%	FP: 14.2%	FN: 11.4%
MCC^c^	0.27		0.36		0.39	
Accuracy	0.64		0.69		0.74	
Precision	0.70		0.68		0.80	
Recall	0.68		0.84		0.83	
F1 Score	0.69		0.75		0.82	
ROC AUC	0.63		0.67		0.69	

a TP: true positives; FP: false positives; TN: true negatives; and FN: false negatives.

b True active compounds were identified according to the predictions.

c MCC values are evaluated based on the predictions made on PC3, DU145, and LNCaP *ext-validation* sets.

The PC3 and DU145 classifiers yielded slightly lower, yet still satisfactory, performance metrics (Table 4), demonstrating their ability to discriminate active from inactive compounds, even when applied to structurally novel molecules.

As expected, predictive performance on the *ext-validation* data sets (Table 4) was lower than that observed during model training (Table 2). To better contextualize these findings, an applicability domain analysis was performed to evaluate the overlap between the chemical spaces represented by the training/test and *ext-validation* data sets. For each compound in the *ext-validation* data sets, nearest-neighbor similarities relative to the training compounds were calculated using both MACCS keys and ECFP4 fingerprints. Compounds were considered within the applicability domain only when both similarity criteria were satisfied (i.e., MACCS similarity ≥0.80 and ECFP4 similarity ≥0.30), in line with commonly accepted thresholds.^42,43^

To further visualize the relationship between training and external data sets, uniform manifold approximation and projection (UMAP) analyses were performed (Figure S5).^44^ These analyses revealed estimated chemical-space overlap values of 54%, 56%, and 5% for PC3, DU145, and LNCaP, respectively. Importantly, a substantial fraction of *ext-validation* compounds occupied peripheral or previously unexplored regions of chemical space relative to the training data sets. These findings suggest that the lower MCC values observed for external validation are attributable, at least in part, to chemical space extrapolation beyond the applicability domain of the models. Because ML classifiers generally perform best on compounds located within or near the chemical space represented during training,^28,29^ the reduced predictive performance observed on the *ext-validation* set is consistent with the limited overlap identified by the applicability domain analyses. More broadly, these results highlight a common limitation of ML applications in drug discovery. Predictive performance estimated from conventional training/test set partitions may overestimate model generalizability when classifiers are applied to structurally novel compounds that can be encountered during prospective virtual screening campaigns.

Collectively, the independent evaluation of LB similarity screening and ML classification highlighted their complementary strengths and limitations. Similarity-based methods effectively enriched active compounds but generated a substantial number of false positives, whereas ML classifiers improved discrimination between active and inactive compounds but exhibited reduced performance when extrapolating beyond the training chemical space. These observations provided the rationale for integrating both methodologies into a unified workflow.

### Integration of Ligand-Based Similarity and ML Classification Approaches

To address these limitations, we integrated both methodologies into a unified workflow. Importantly, LB similarity filtering was not employed as an additional prioritization strategy. Rather, it was explicitly incorporated as a chemical space-focusing step designed to constrain ML predictions to compounds located closer to the applicability domain of the models. The rationale underlying this approach was that compounds structurally related to known actives are more likely to reside within regions of chemical space where ML predictions are expected to be more reliable.

The performance of the resulting *combined approach* was evaluated using the same external validation sets employed throughout this study. In line with previous comparisons, *Precision* was chosen as the primary metric for evaluating these improvements. As shown in Figure 2 and Tables S4 and Table S5, the *Combined approach* substantially improved screening performance relative to either approach applied independently. The most evident benefit was a marked reduction in false-positive predictions, which translated into higher *Precision* (Figure 2 and Table S4). This improvement was particularly relevant for LNCaP, which exhibited the highest false-positive rate in the LB screening and the lowest overlap between training and external chemical spaces according to the applicability domain analyses. In this case, *Precision* improved from 0.48 for similarity screening alone to 0.61 after application of the *Combined approach* (Table S4). The advantages of the *Combined approach* became even more evident when progressively restricting the analysis to the highest-ranked compounds identified by similarity screening. Within the top 10% of the similarity rankings, the *Combined approach* achieved *Precision* values of 1.0, 0.9, and 0.8 for LNCaP, PC3, and DU145, respectively (Figure 2). These results demonstrate that ML classification was particularly effective in refining candidate prioritization after an initial similarity-based enrichment step.

**Figure 2:**
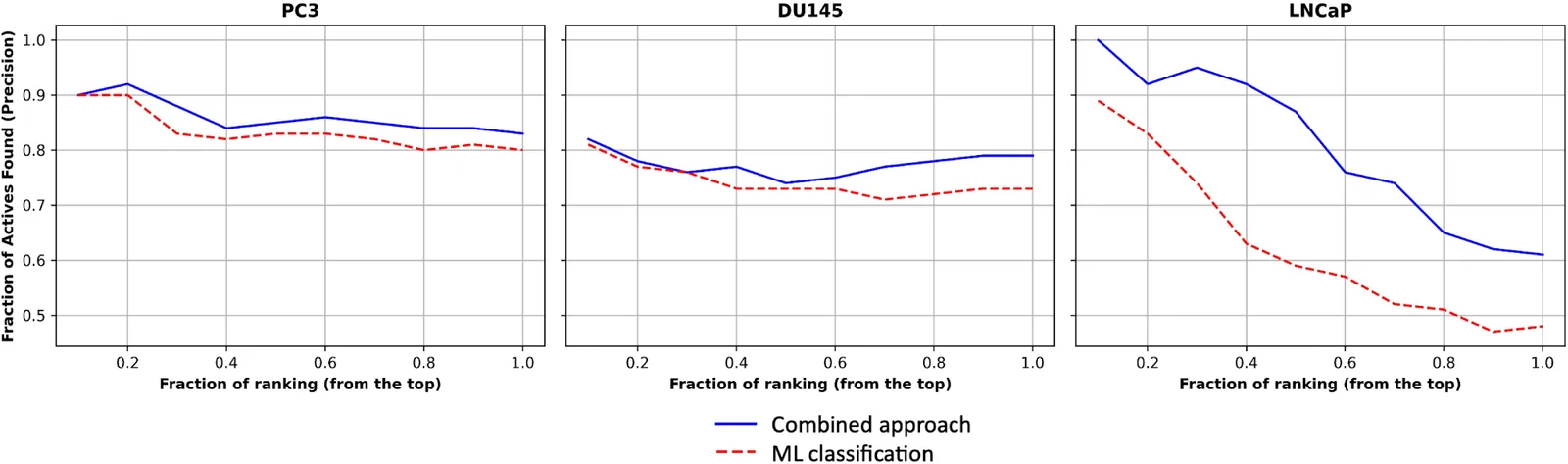
Comparison of the precision curves of the ML classifiers and of the *Combined approach*. For each *ext-validation* set, the compounds were ranked according to the number of similar molecules with antiproliferative activity (10 μM threshold) against PC3, DU145, or LNCaP training sets, according to MACCS*fp*- and ECFP4*fp*-based Tanimoto indeces (Supporting File 1). The blue and dashed red lines report the *Precision* trend of the combined approach and ML classifier alone, respectively, considering only the top percentage of the similarity rank.

To further evaluate early recognition performance, enrichment factors were calculated at the top 1%, 2%, 5%, and 10% of the similarity rankings (Table 5). Across all three data sets, the *Combined approach* consistently outperformed similarity screening alone. In line with *Precision* results (Figure 2), the largest improvements were observed for LNCaP, supporting the hypothesis that applicability domain-guided filtering can be especially beneficial when screening chemically diverse data sets.

**Table 5:** Top 1%, 2%, 5%, and 10% Enrichment Factors Evaluated for the Similarity and Combined Approaches, Applied to the PC3, DU145, and LNCaP *Ext-Validation* Datasets

	PC3		DU145		LNCaP	
	LB similarity	Combined approach	LB similarity	Combined approach	LB similarity	Combined approach
Top 1% enrichment	1.5	1.9	1.4	1.8	1.8	2.3
Top 2% enrichment	1.5	1.8	1.4	1.6	1.8	2.2
Top 5% enrichment	1.4	1.6	1.6	1.8	1.8	2.2
Top 10% enrichment	1.6	1.6	1.5	1.7	1.7	1.8

The increased *Precision* of the combined workflow is highly relevant for prospective drug discovery, as it reduces experimental resources spent on false positives. Consequently, this approach not only improves predictive performance but also enhances the overall efficiency of prospective virtual screening campaigns. To the best of our knowledge, previously reported workflows generally apply LB similarity methods and ML models either independently or as parallel prioritization strategies. In contrast, the present workflow explicitly uses similarity information to improve compliance with the applicability domain of ML classifiers before prediction. The resulting strategy reduces unreliable chemical space extrapolation and improves the robustness of candidate prioritization.

### Selection of Drug Repurposing Candidates and *In Vitro* Testing on PC3-Derived Multicellular Tumor Spheroids (MTSs)

The validated *combinedapproach* was subsequently applied to identify the most promising drug repurposing candidates for *in vitro* testing. Ligand-based virtual screening was performed on the DrugBank compounds analyzed in our previous work.^33^ The application of the developed classifiers predicted antiproliferative activity for 62, 45, and 27 compounds in the DU145, PC3, and LNCaP models, respectively. Notably, 16 compounds were consistently predicted to be active across all three PCa cell lines (Table S6), highlighting them as particularly promising candidates for biological evaluation. Of these, 11 drugs belonging to well-established anticancer drug classes (*e.g*., classical antifolates and anthracyclines) were excluded. The remaining five drugs (BMS-2, 5-iodotubercidin, olomoucine II, digoxigenin, and chlormidazole) were selected for *in vitro* biological testing.

Initial biological evaluation was performed using PC3-derived multicellular tumor spheroids (MTSs), which provide a physiologically relevant three-dimensional model of tumor growth. To promote 3D growth, PC3 cells were embedded in 0.5 mg/mL Matrigel to form spheroids and subsequently treated with each compound at a 20 μM concentration; this concentration, which is higher than that used in 2D cultures, was selected to compensate for the reduced drug diffusion and cellular uptake typically associated with 3D spheroid models while still avoiding toxic or clinically irrelevant conditions. This approach enabled the rapid identification of the most promising candidates during this preliminary 3D screening phase. Spheroid morphology was monitored by optical microscopy (Figure 3, panel A), and spheroid area (normalized to day 0) was measured over 9 days and compared to untreated control cells (Ctr) (Figure 3, panel B). Control spheroids showed a gradual increase in size, with a compact core and an outer ring of proliferative cells. In contrast, four of the five compounds (BMS-2, 5-iodotubercidin, olomoucine II, and chlormidazole) induced a marked inhibition of spheroid growth relative to untreated controls (Figure 3, panels A, B), whereas digoxigenin caused a progressive increase in relative area over time. However, this effect is likely due to time-dependent drug-induced disaggregation of the MTSs rather than increased cell proliferation.

**Figure 3:**
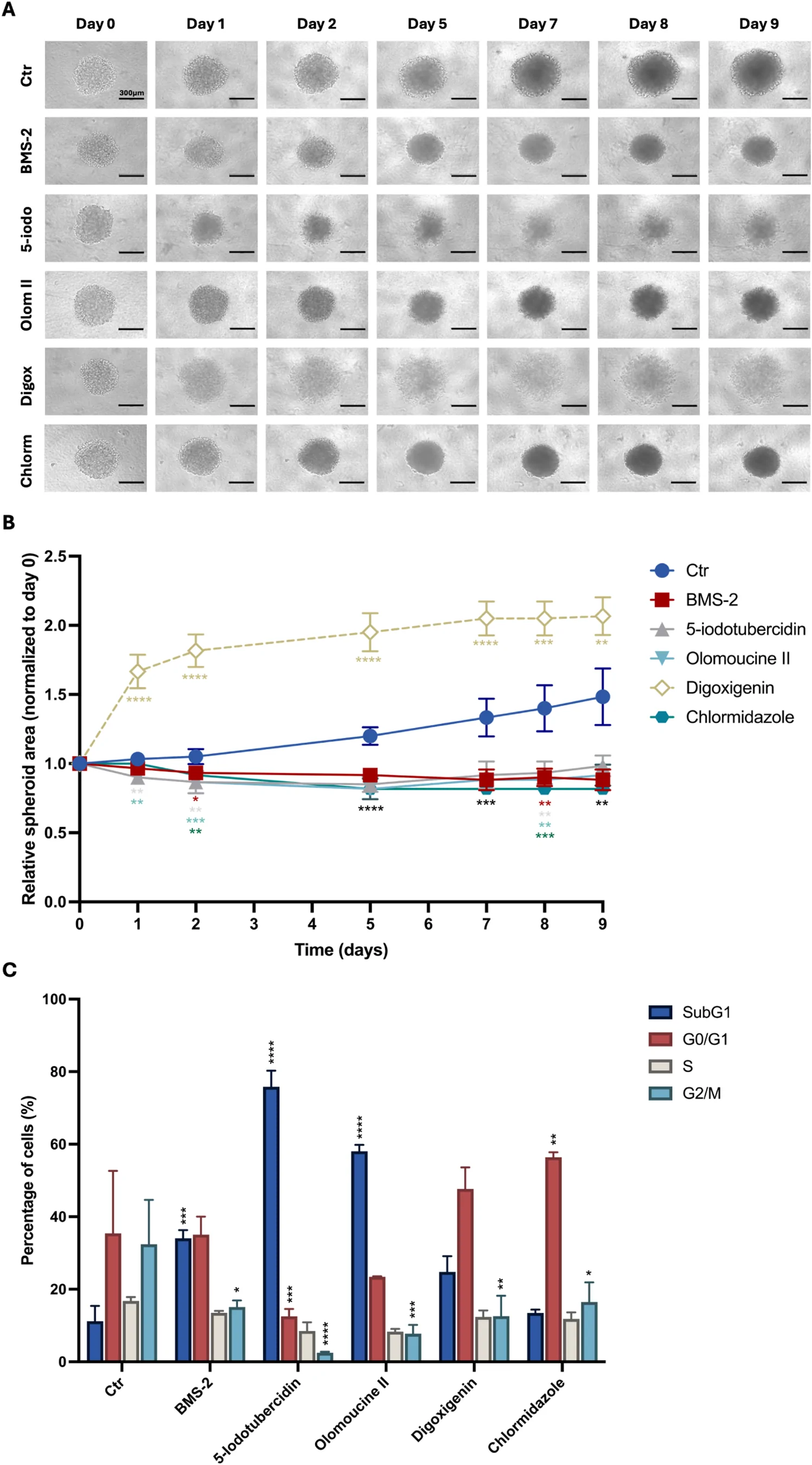
Antiproliferative effect of the five most promising compounds (i.e., BMS-2, 5-iodotubercidin, olomoucine II, digoxigenin, and chlormidazole) on PC3 multicellular tumor spheroids (MTSs). Spheroids were treated with compounds at a 20 μM concentration and monitored daily, from day 0 (immediately post-treatment) to day 9. Panel (A) reports representative phase-contrast microscopy images of PC3 MTSs from day 0 to day 9, either untreated (control, Ctr) or treated with compounds. Scale bar = 300 μm. Panel (B) reports the relative area (mean ± SD) normalized to day 0 of both treated and untreated (Ctr) MTSs, determined daily from day 0 to day 9. Asterisks indicate statistical significance between the treated groups vs the Ctr group (untreated MTSs) for each day. For each compound, statistical significance is indicated by the color of the respective sample; when significance is consistent across all compounds except digoxigenin, black asterisks are used. Panel (C) shows the effects of compound treatment on cell-cycle progression in PC3 cells within MTSs, determined at day 9. Data (mean ± SD) are expressed as the percentage of PC3 cells across the different cell cycle phases (two-way ANOVA with Dunnett multiple comparison test. Panel A and B analyses: *n* = 6; panel C analyses: *n* = 2 independent analysis on a pool of three spheroids that were independently generated and independently treated, resulting in a total of *n* = 6 independent spheroids for each condition. *p < 0.05, **p < 0.01, *** *p* < 0.001, ****p < 0.0001).

To further characterize the observed phenotypes, cell cycle analyses were performed on MTSs after 9 days of treatment. BMS-2, 5-iodotubercidin, and olomoucine II showed a marked increase in the SubG1 population and a significant reduction in the G2/M fraction compared to untreated MTSs (Ctr), indicating a cytotoxic effect. Although not statistically significant, an increase in the sub-G1 fraction was also observed in digoxigenin-treated cells, accompanied by a significant decrease in the G2/M phase. In contrast, chlormidazole induced a significant G0/G1 accumulation together with a reduction of G2/M population without evidence of apoptosis, consistent with a cytostatic mechanism of action (Figure 3, panel C).

Collectively, these results demonstrate that the *combined approach* successfully identified multiple biologically active repurposing candidates.

### *In Vitro* Activity in 2D PCa and Primary Prostate Cell Models

Given the promising antiproliferative activity observed in MTSs, the five candidates were further evaluated for their antiproliferative effects in 2D cultures *in vitro*. Specifically, androgen-responsive (LNCaP) and nonresponsive (PC3) PCa cells cultured in 2D were treated to determine the GI_50_ dose, defined as the drug concentration required to inhibit cell growth by 50%. These experiments also provided an opportunity to assess the ability of the workflow to identify active compounds across biologically distinct prostate cancer models. After 72 h of treatment, all five compounds demonstrated marked antiproliferative activity, with GI_50_ values in the single-digit micromolar range, thereby confirming their potential as repurposing candidates (Table 6). These findings independently confirmed the results in the 3D spheroid model and further supported the predictive ability of the adopted workflow.

**Table 6:**
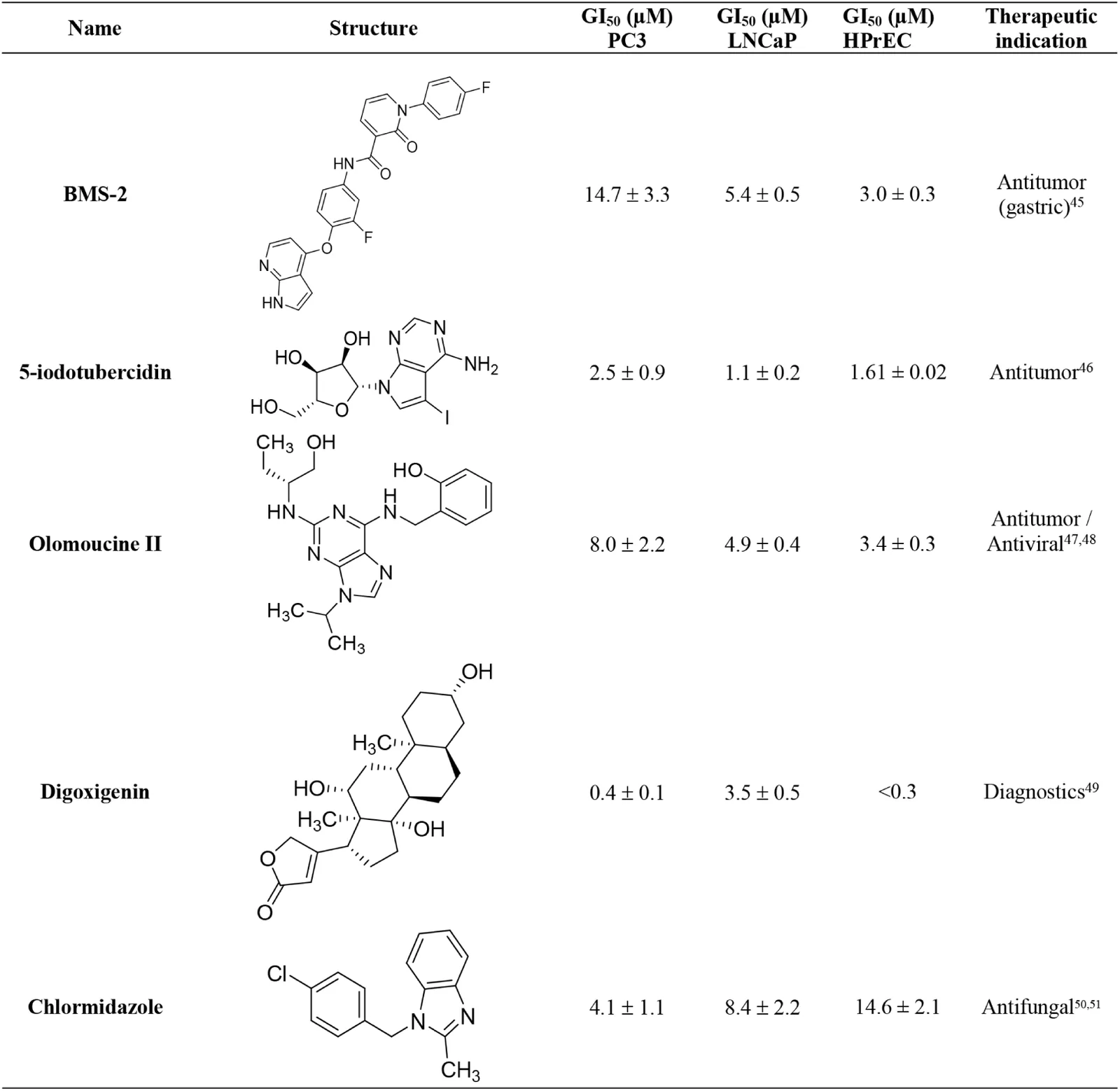
Antiproliferative Activity (GI_50_, Mean ± SEM, μM) of the Selected Drug Repurposing Candidates on PC3 and LNCaP PCa Cells, as Well as on Healthy Primary HPrEC Cells, after 72 H Treatment, along with Their Primary Therapeutic Indications^45,46,47,48,49^

To evaluate cancer cell selectivity, GI_50_ values were also measured in normal primary prostate epithelial cells (HPrEC). Among the tested compounds, chlormidazole showed marked tumor selectivity, with GI_50_ values approximately 3-fold higher in HPrEC (GI_50_ = 14.6 ± 2.1 μM) than in PC3 cells (GI_50_ = 4.1 ± 1.1 μM), and roughly 2-fold higher than in LNCaP cells (GI_50_ = 8.4 ± 2.2 μM). This selectivity profile distinguished chlormidazole from the other candidates and identified it as the most promising candidate for further investigation.

Notably, the combination of activity across androgen-responsive and androgen-independent PCa models, efficacy in both 2D and 3D cellular systems, and preferential activity toward malignant cells represent a particularly attractive profile for a repurposing candidate. For this reason, subsequent mechanistic and biological studies focused on chlormidazole.

### Mechanistic Characterization of the Cytostatic Activity of Chlormidazole

To better characterize the biological effects of chlormidazole, cell-cycle analyses were performed in PC3 and LNCaP cells following 72 h of treatment. A propidium iodide-based assay was first conducted, as performed for MTSs. In both cell lines, chlormidazole induced a dose-dependent increase in the G0/G1 population (Figure 4, left panels A and B), accompanied by either a decrease in the G2/M fraction in PC3 cells or a decrease in the S-phase fraction in LNCaP cells. These results were further supported by BrdU incorporation assays, which showed a reduction in S-phase cells, reaching statistical significance in LNCaP cells only (Figure 4, right panels A and B). Importantly, no apoptotic events were detected even at the highest concentrations tested.

**Figure 4:**
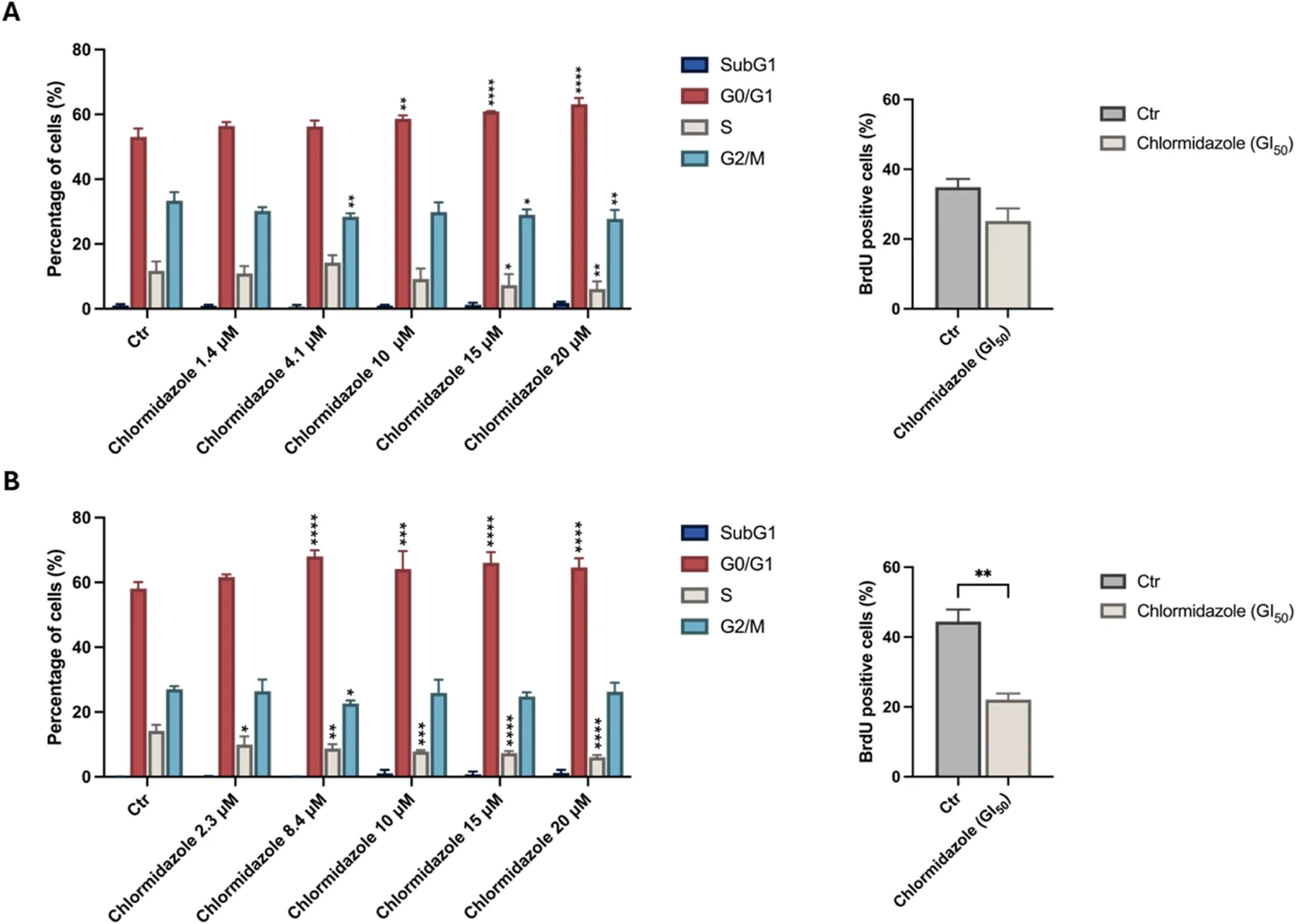
Dose–response effects of chlormidazole on cell-cycle progression in PCa cells. Data represent the percentage of PC3 (left panel A) and LNCaP (left panel B) cells across the different cell-cycle phases monitored through Propidium Iodide staining after 72 h of treatment. Data are reported as mean ± SD. Data from the BrdU-based assay on PC3 (right panel A) and LNCaP (right panel B) cells at GI_50_ doses after 72 h of treatment represent the percentage of cells positive to BrdU incorporation. Data are reported as mean ± SEM (left panel A, B: two-way ANOVA with Dunnett multiple comparison test, *n* = 3; right panel A, B: Student’s *t*-test, *n* = 3; *p < 0.05, **p < 0.01, *** *p* < 0.001, ****p < 0.0001).

Given the pronounced cytostatic phenotype induced by chlormidazole, additional studies were performed to investigate the molecular pathways potentially involved in its antiproliferative activity. As an initial step, we evaluated whether chlormidazole directly inhibited kinase complexes known to regulate the G1/S transition. *In vitro* assays were performed against CDK2/CycE, CDK4/CycD1, CDK6/CycD1, PI3Kα (p110a/p85a), PI3Kβ (p110b/p85a), AKT1, and mTOR/FRAP1. No significant direct inhibition of these targets was detected, suggesting that the observed effect is unlikely to result from direct blockade of these major proliferative signaling nodes. To further investigate the molecular basis of the cytostatic response, gene and protein expression analyses were performed focusing on key regulators of cell-cycle progression. Because the antiproliferative phenotype was observed in both LNCaP and PC3 cells, we sought molecular alterations shared by both models. The involvement of p53 appeared unlikely, given the absence of p53 in PC3 cells and the lack of significant p53 induction in LNCaP cells. While CyclinB1 transcript levels were reduced, in line with a reduction in the percentage of G2/M phase cells, no significant changes were observed in CyclinD1 expression in both LNCaP and PC3 cells (Figure 5, panel A and Figure S6). RT-qPCR analyses identified p57 as the only CDK inhibitor significantly induced by chlormidazole treatment, while no appreciable changes were observed in the expression of p16 or p21. Consistent with the transcriptional data, p57 was also upregulated at the protein level. Importantly, p57 upregulation was accompanied by reduced phosphorylation of the retinoblastoma protein (Rb), a central regulator of G1/S progression and a major downstream target of CDK4/6 activity, thereby supporting G1 cell-cycle arrest (Figure 5, panel B). These findings are consistent with the maintenance of Rb in its growth-suppressive state and provide a mechanistic explanation for the G1 accumulation described above. In addition, chlormidazole treatment did not induce transcriptional activation of the pro-apoptotic Bax and Noxa genes, consistent with a cytostatic rather than genotoxic effect. Likewise, no evidence of DNA damage was detected, as shown by the absence of γ-H2AX (phospho-Ser139) accumulation in Western blot analyses.

**Figure 5:**
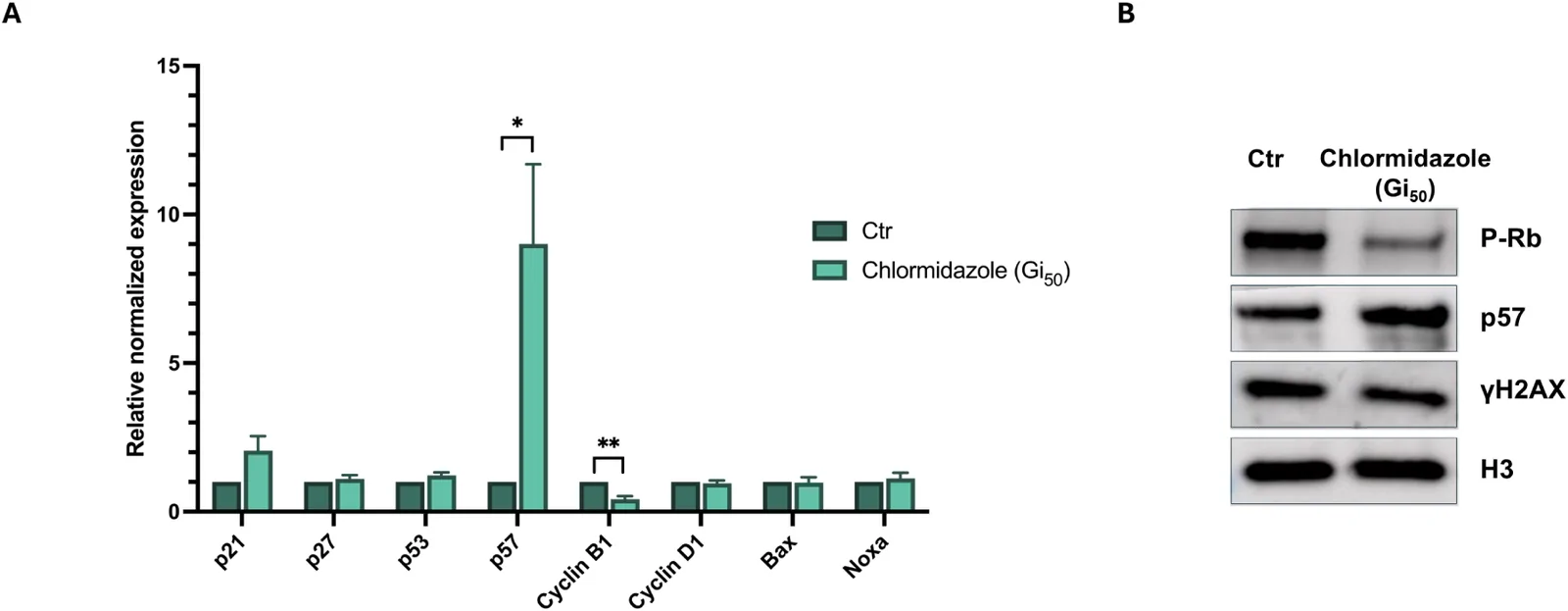
Panel A: RT-qPCR analysis of the indicated transcripts in LNCaP cells after 72 h of treatment with chlormidazole at the GI_50_ dose. RPS20 was used as the reference gene, and normalized mRNA levels are reported as fold change vs untreated cells (Ctr), arbitrarily set at 1. Data represent mean ± SEM (Student’s *t* test, *n* = 3: *p < 0.05, ** *p* < 0.01). Panel B: Western blot analysis of phospho-Rb, p57, and γH2AX protein expression in LNCaP cells treated with chlormidazole for 72 h. Histone H3 was used as the loading control.

Overall, these transcriptional and protein expression data support a model in which chlormidazole induces a predominantly cytostatic phenotype associated with p57 upregulation, reduced Rb phosphorylation, and inhibition of cell-cycle progression in the G1 phase without activation of apoptosis or DNA damage. Although the direct molecular target(s) of chlormidazole remain unknown, the present results provide mechanistic evidence linking compound exposure to activation of a p57-associated G1 cell-cycle arrest program.

### *In Vitro* Evaluation of the Chlormidazole-Docetaxel Combination in PCa and Primary Prostate Cells

Chlormidazole (DrugBank ID: DB13611, CHEMBL ID: CHEMBL152649) is a benzimidazole antifungal agent that inhibits ergosterol biosynthesis in fungi.^50,51,52,53,54,55^ Azole-containing drugs have been investigated for a wide range of therapeutic applications, including antiviral, antiulcer, antihypertensive, antiparasitic, and anticancer uses.^56,57,58,59^ A notable example is ketoconazole, an established antifungal drug that has undergone clinical evaluation in combination with docetaxel for PCa treatment.^60,61,62,63^ To provide a clinically relevant benchmark, the antiproliferative activity of ketoconazole was evaluated for comparison. Ketoconazole was approximately 8-fold less potent than chlormidazole in PC3 cells (GI_50_ = 31.8 ± 2 μM) and 2-fold less potent in LNCaP cells (GI_50_ = 16.1 ± 3.2 μM) (Figure S7). In addition, ketoconazole lacked tumor cell selectivity, showing higher potency toward healthy prostate epithelial cells (HPrEC; GI_50_ = 1.5 ± 0.3 μM) than PCa cells (Figure S7). Together, these findings further support the prioritization of chlormidazole as the most potent and tumor-selective therapeutic candidate.

To further explore its therapeutic potential, we next investigated the potential of chlormidazole in combination with docetaxel, which remains a key treatment for advanced PCa. A dose–response assay was performed in PC3 cells using decreasing concentrations of docetaxel, either alone or in combination with a fixed concentration of chlormidazole corresponding to its experimentally determined GI_30_ (1.4 ± 0.2 μM) (Figure 6, left panel A). Co-treatment with chlormidazole significantly enhanced the antiproliferative activity of docetaxel, reducing its GI_50_ from 2.0 ± 0.4 nM to 0.8 ± 0.2 nM (Figure 6, right panel A). The combination experiment was also conducted in primary noncancerous prostate epithelial cells (HPrEC; Figure 6, left panel B) using the same fixed chlormidazole concentration, which falls within the HPrEC GI_30_ confidence interval (GI_50_ = 2.2 ± 0.8 μM). In contrast to the results observed in PC3 cells, the presence of chlormidazole increased the GI_50_ of docetaxel in HPrEC cells from GI_50_ = 0.8 ± 0.1 nM to GI_50_ = 2.5 ± 0.1 nM, indicating that the combination reduces docetaxel toxicity toward noncancerous prostate cells (Figure 6, right panel B). Collectively, these findings show that chlormidazole increases docetaxel potency in PCa cells while simultaneously reducing its toxicity toward healthy prostate cells. From a translational perspective, this observation is particularly relevant because it suggests that comparable biological activity may be achieved using lower (less than half) concentrations of docetaxel, potentially reducing its well-documented dose-limiting adverse effects^64,65^ while simultaneously reducing toxicity toward noncancerous prostate cells.

**Figure 6:**
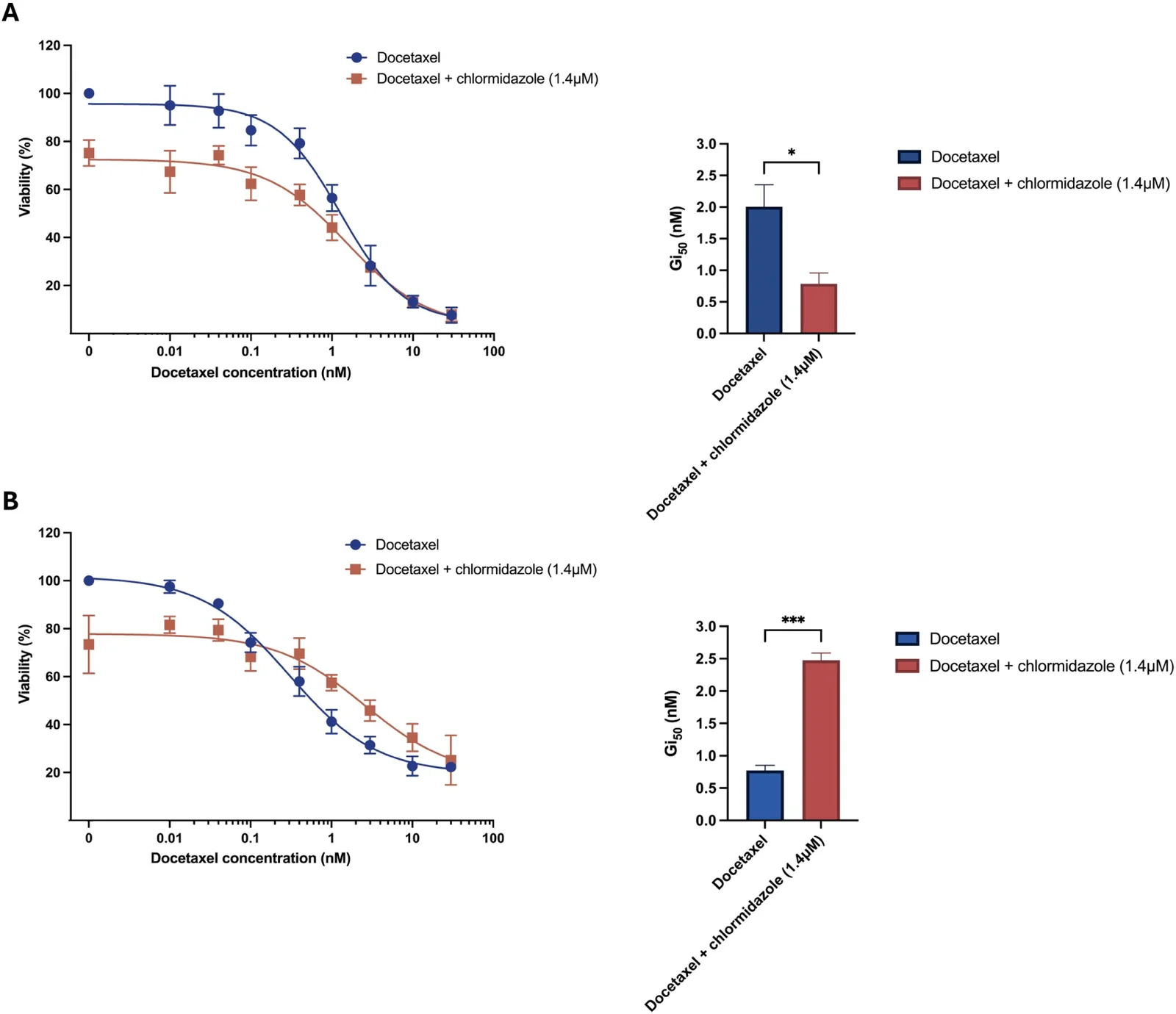
Effect of docetaxel alone or in combination with chlormidazole in PC3 and HPrEC cells. Dose–response curves in PC3 (left panel A) and HPrEC (left panel B) cells, obtained after incubation for 72 h with docetaxel alone or in combination with chlormidazole. The curves were generated starting from a docetaxel concentration of 30 nM, followed by 3-fold serial dilutions up to 0.014 nM, alone or in combination with a fixed concentration of chlormidazole (1.4 μM). Right panel A and right panel B show the difference in GI_50_ of docetaxel alone or combined with chlormidazole in PC3 and HPrEC cells, respectively. Data represent mean ± SEM (Student’s *t* test, *p < 0.05; ***p < 0.001; *n* = 3).

To determine whether these results could be reproduced in a more physiologically relevant cancer model, combination studies were extended to PC3-derived MTSs. As an initial step, MTSs were exposed to increasing concentrations of docetaxel, and antiproliferative effects were assessed by comparing each spheroid to its own baseline size (day 0, immediately after treatment) and calculating the relative reduction in size after 10 days of treatment. Dose–response experiments using 0.8, 1.3, and 2 nM docetaxel showed that the 2 nM concentration (GI_50_ dose) reduced spheroid size by approximately 50% (Figure 7, panel A). The same approach was applied to chlormidazole, confirming the results obtained in 2D cultures: treatment with 4.1 μM chlormidazole (GI_50_ dose) decreased spheroid size by about 60% (Figure 7, panel B). In line with the combination studies performed in 2D cultures, suboptimal concentrations corresponding to the GI_30_ values of both agents were then selected for further evaluation in 3D MTSs. Co-administration of 1.3 nM docetaxel with 1.4 μM chlormidazole resulted in an additional 28% reduction in spheroid size compared with either drug alone, indicating that cotreatment with suboptimal concentrations of chlormidazole and docetaxel produced greater inhibition of spheroid growth than either agent administered alone (Figure 7, panel C). These findings demonstrate that the beneficial effects of the combination are maintained in 3D tumor models and support further investigation of this therapeutic strategy. Overall, these results identify chlormidazole as a promising partner for docetaxel-based therapy and provide a strong rationale for future preclinical studies aimed at evaluating the translational potential of this combination in advanced PCa.

**Figure 7:**
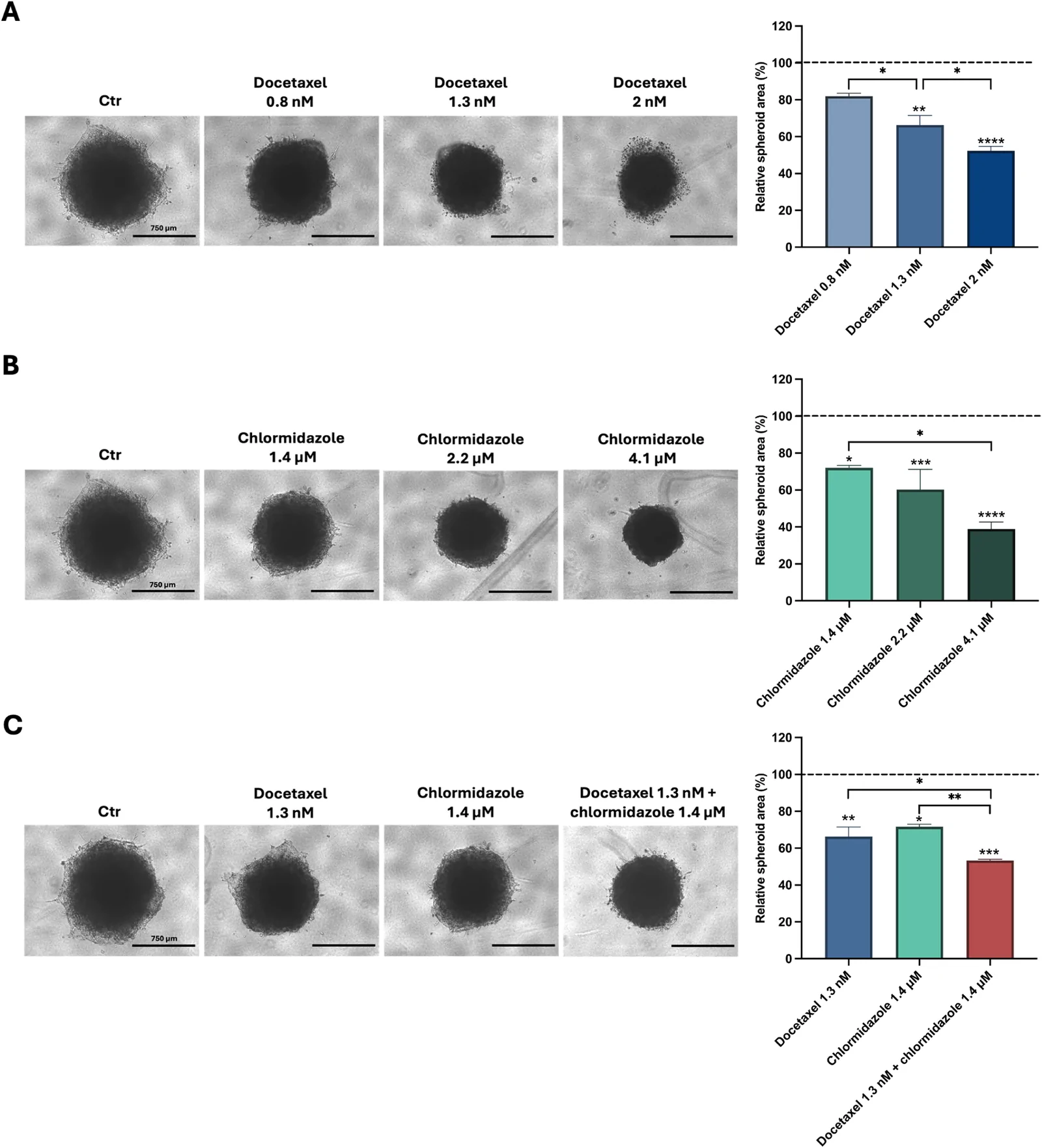
Effect of docetaxel, chlormidazole, and their combined treatment on PC3MTSs. Panel A: representative phase-contrast microscopy images of MTSs treated for 10 days with docetaxel at 0.8, 1.3, and 2 nM concentrations (left). Scale bar = 750 μm. The corresponding effects on spheroid size relative to untreated controls are quantified (right). Panel B: representative phase-contrast microscopy images of MTSs treated for 10 days with chlormidazole at 1.4, 2.2, and 4.1 μM concentrations (left). The histogram shows the reduction in spheroid growth compared to untreated MTSs (right). Scale bar = 750 μm. Panel C: representative phase-contrast microscopy images of MTSs treated for 10 days with 1.3 nM docetaxel and 1.4 μM chlormidazole, alone or in combination (left). Scale bar = 750 μm. The effect on MTS growth relative to untreated spheroids is reported (right). Data represent mean ± SEM (one-way ANOVA with Tukey multiple comparison test (comparison among treatments) or Dunnett multiple comparison test (treatments vs Ctr comparison), *n* = 3; *p < 0.05, **p < 0.01, *** *p* < 0.001, ****p < 0.0001).

## Conclusions

In this study, we developed and prospectively evaluated an integrated computational-experimental workflow that combines ligand-based similarity screening with machine-learning classification for drug repurposing applications in prostate cancer. We found that predictive performance estimated from conventional training/test set partitions may substantially overestimate performance metrics and model generalizability when classifiers are applied to structurally novel compounds. Evaluation on independent external data sets yielded suboptimal predictive performance relative to internal validation. These findings highlight the importance of explicitly considering applicability-domain coverage when assessing ML models for prospective virtual screening applications. To address this critical issue, we implemented a combined strategy that integrates ligand-based similarity methods with ML models. By restricting predictions to compounds located closer to the applicability domain of the models, the *Combined approach* markedly reduced false-positive rates, improved hit enrichment, and increased *Precision* across all external validation data sets. The resulting workflow is broadly applicable and well-suited for high-throughput virtual screening and drug-repurposing campaigns.

Prospective application of the validated computational workflow to DrugBank compounds led to the identification of five experimentally confirmed repurposing candidates with antiproliferative activity against both androgen-sensitive and -insensitive PCa cells. All five compounds exhibited marked antiproliferative effects across both 3D and 2D cellular models, consistent with the computational predictions. Among them, chlormidazole emerged as the most promising drug, displaying activity in both androgen-responsive and -independent prostate cancer cells, efficacy in 3D spheroid models, and a favorable selectivity profile toward malignant cells versus primary noncancerous prostate cells. Mechanistic investigations indicated that chlormidazole exerts a predominantly cytostatic effect associated with the activation of the CDK inhibitor p57 and reduction of phosphorylated RB levels, consistent with cell-cycle inhibition rather than induction of cell death or DNA damage. Although the direct molecular target(s) of chlormidazole remain to be identified, the present study establishes a mechanistic framework that can guide further target deconvolution studies.

Notably, chlormidazole displayed significantly greater efficacy and lower toxicity than ketoconazole, an antifungal drug that has undergone clinical evaluation in combination with docetaxel for PCa treatment, further highlighting its considerable translational potential. Importantly, when combined with the standard chemotherapeutic agent docetaxel, chlormidazole reduced docetaxel toxicity toward healthy prostate cells while enhancing its antiproliferative activity in both 2D and 3D PCa models. This combination enabled substantially lower docetaxel doses to achieve comparable therapeutic efficacy. Such a dose reduction is clinically relevant because it may mitigate the well-documented adverse effects associated with docetaxel treatment while potentially limiting the emergence of drug resistance. Altogether, this work identifies chlormidazole as a promising repurposing candidate for prostate cancer while also providing a broadly applicable framework for improving the reliability of machine-learning-assisted virtual screening and drug repurposing campaigns.

## Experimental Section

### Computational Studies

#### Data Set Preparation

In a previous study,^33^ we identified several DrugBank compounds structurally similar to ChEMBL molecules (release 29, accessed on November 2021) with reported antiproliferative activity against PCa cell lines. Ninety of these compounds had never been tested against prostate cancer, suggesting a potential drug repurposing application. In the present work, we integrated these results with machine learning classification to select a focused subset of DrugBank compounds for experimental testing. To ensure robust ML model development, both active and inactive compounds were collected from ChEMBL29 (https://www.ebi.ac.uk/chembl/; release 29; accessed on November 2021) and the more recent ChEMBL34 release (https://www.ebi.ac.uk/chembl//; release 34; accessed on October 2024).^9^ Data curation involved strict filtering, as detailed in our recent article.^31^ Compounds that passed the filtering process were labeled as “active” or “inactive” based on a 10 μM threshold, as done previously,^33^ and in line with previous literature data.^35^

The adopted filtering protocol yielded 10,505 compounds for PC3, 9004 for DU145, and 3002 for LNCaP cells from the ChEMBL29 data set (see Supporting File 4), which were used to develop ML classifiers. Filtering of ChEMBL34 produced 12,766 compounds for PC3, 9869 for DU145, and 3444 for LNCaP. After removing molecules already present in ChEMBL29, 2292 compounds for PC3 (1406 actives, 886 inactives), 1054 for DU145 (606 actives, 448 inactives), and 464 for LNCaP cells (320 actives, 144 inactives) were obtained. These data sets, hereafter referred to as the external validation (“*ext-validation*”) data sets were used to validate the integrated chemoinformatics/machine-learning workflow described in this study (Supporting File 5). The generated ChEMBL29 and *ext-validation* data sets were subsequently prepared as follows. The Pandas (https://pandas.pydata.org/) and RDKit (https://www.rdkit.org/) Python libraries were employed for molecular standardization, including the generation of canonical SMILES, addition of hydrogen atoms, desalting, and assignment of formal charges. For each compound, 220 molecular descriptors were calculated with RDKit (Supporting Files 4 and 5). The same molecular descriptor generation protocol was applied to the DrugBank compounds (Supporting File 6).

#### Similarity-Based Screenings

Similarity estimations were carried out using ECFP4 and MACCS fingerprints implemented in the RDKit (https://www.rdkit.org/) Python library with default settings. ChEMBL compounds with reported antiproliferative activity against selected PCa cell lines were used as queries (Supporting Information 1), following the fingerprint-based similarity screening approach described in our previous study.^33^ Similarity thresholds were set to 0.8 for MACCS and 0.3 for ECFP4, in line with commonly accepted standards.^42,43^

#### Development and Validation of the Machine-Learning Classifiers

Machine-learning classifiers were developed with the Scikit-Learn modules implemented in Python (*Scikit-Learn version 1.3.2*).^37^ Descriptors containing missing or non-numeric values were removed prior to model development. Then, data sets were split into training and test sets with a 70:30 ratio. To ensure a correct representation of all chemical structures, we implemented a custom clustering-based splitting strategy through *in-house* Python scripts. Molecules with a Tanimoto similarity ≥0.8 (MACCS fingerprints) were grouped together and then evenly partitioned among the training and test sets. Active/inactive class balance was also ensured (Figures S1–S3) to avoid biases that could negatively affect model development and performance.^66^

After the initial descriptor curation, all model-dependent preprocessing steps were performed on the training set. In particular, descriptors with near-zero variance or high pairwise correlation were removed, and the remaining ones were standardized using the RobustScaler method implemented in Scikit-Learn. Afterward, ML classifiers were built with the Extra Trees algorithm (*sklearn.ensemble.*ExtraTreesClassifier) according to the following procedure: (i) tuning of the hyperparameters via a 10-fold stratified cross-validation procedure with the *GridSearch* CV module implemented in Scikit-Learn; (ii) elimination of redundant features with a 10-fold stratified cross-validation procedure through RFECV; (iii) hyperparameter tuning on the reduced descriptor space; (iv) training of the models; (v) evaluation of model performance on the test set using multiple metrics. All cross-validation procedures were performed using StratifiedKFold, preserving the distribution of active and inactive compounds across folds. During RFECV, molecular descriptors were iteratively removed according to their contribution to model performance using the Extra Trees classifier as an estimator and MCC as an optimization metric. Hyperparameters used for optimization are listed in Table S1, while selected features after RFECV are reported in Table S2. Model performance was evaluated through widely adopted metrics, including *MCC*, *accuracy*, *precision*, *recall*, *F1-score*, and *ROC AUC*. MCC was selected as the primary evaluation metric because of its robustness in binary classification tasks and its ability to simultaneously account for true positives, true negatives, false positives, and false negatives.^39^ The trained ML classifiers were then applied to the *ext-validation* sets. Comparative analysis of true positives, false positives, false negatives, and true negatives, along with comparison of precision and ROC-AUC curves, was then performed.

#### Analysis of Model Applicability Domain

Applicability domain analysis was performed to evaluate the overlap between the chemical space represented in the training and external validation data sets. Pairwise molecular similarities between external and training compounds were calculated using MACCS keys and ECFP4 fingerprints. For each external compound, the nearest-neighbor similarity to the training set was determined. Compounds were considered within the applicability domain only when both similarity criteria were satisfied, namely, MACCS similarity ≥0.80 and ECFP4 similarity ≥0.30,^42,43^ relative to at least one training compound. To visualize the overlap between data sets, similarity matrices were projected into a low-dimensional chemical space using UMAP with default settings,^44^ by means of a custom python script. External compounds were subsequently classified as inside or outside the AD and visualized together with the training compounds.

#### Selection of DrugBank Candidates

The validated ML models were applied to the DrugBank compounds (Supporting Information 6) identified in our earlier work.^31^ Compounds were prioritized and tested *in vitro* against prostate cancer cell lines, as described below. The compounds were purchased from TargetMol (chlormidazole, Lot. ID: 151512), BLD Pharmatech GmbH (BMS-2, Lot. ID: DPA768), 5-iodotubericin (Lot. ID: DNY591) and omoleucine II (Lot. ID: DQZ301), acofarma (Ketoconazole, Lot. ID: 240909-F-2), and biosynth (Digoxigenin, Batch ID: 445221401). All compounds are >95% pure by HPLC analysis, as declared by the vendor.

### Biological Studies

#### Cell Lines

The human prostate cancer cell line PC3 (ATCC Cat# CRL-1435) was cultured in Ham’s F12 (Biowest, Nuaillé, France) medium, and the human prostate cancer cell line LNCaP (ATCC Cat# CRL-1740) was cultured in RPMI 1640 (Biowest, Nuaillé, France) medium. All media were supplemented with 10% Fetal Bovine Serum (FBS, Gibco, Fisher Scientific Italia, Segrate, Italia), 2 mM glutamine, 100 U/ml penicillin, and 100 μg/mL streptomycin. The primary prostate epithelial cells HPrEC (ATCC Cat#PCS-440-010) were grown in Prostate Epithelial Cell Basal Medium (ATCC Cat #PCS-440-030) supplemented with the Prostate Epithelial Cell Growth Kit (ATCC Cat#PCS-440-040). All cells were maintained at 37 °C in a humidified atmosphere of 5% CO_2_.

#### 3D Multicellular Tumor Spheroids Generation and Treatment

5000 PC3 cells/well were seeded into 96-well Round Bottom Ultra-Low Attachment (ULA) plates (Corning #7007 and #4515, 75 μL medium/well). The plates were then centrifuged at 1000*g* for 10 min and incubated at 37 °C with 5% CO_2_ in a humidified environment overnight. The next day, ice-cold Corning Matrigel Basement Membrane Matrix (Corning #354248) was added to each well to reach a final concentration of 0.5 mg/mL. After 24 h of incubation, spheroids were treated with the compounds at a final concentration of 20 μM. Spheroids grown in the cell medium only were used as controls (Ctr). The MTSs were maintained for 9 days at 37 °C, 5% CO_2_ in a humidified incubator. Every 3 days, each well was refilled with fresh solutions of the drugs (20 μM) in cell medium or cell medium only (Ctr). Images of the MTSs were captured from day 0 (immediately post-treatment) to day 9 using the EVOS M5000 imaging system (Thermo Fisher Scientific). The acquired images were analyzed with ImageJ software to calculate the total spheroid area. The relative spheroid area, normalized to day 0, was determined for each time point using the following formula: Relative Spheroid Area = Spheroid Area at day *n*/Spheroid Area at day 0.

#### Cell Cycle Analyses of MTSs by Flow Cytometry

Quantitative measurements of the cell-cycle phase distribution of PC3 cells assembled in 3D spheroids were performed by flow cytometry. Briefly, MTSs were disaggregated by incubation with 1X Trypsin at 37 °C, using gentle pipetting to facilitate dissociation. Afterward, cells were suspended in 0.5 mL of propidium iodide solution (50 μg/mL propidium iodide, 3.4 mM sodium citrate, 0.1% Triton X-100 in PBS) and analyzed by an Attune NxT Flow Cytometer.

#### 2D Cell Viability Assays

PC3, LNCaP, and HPrEC cells were seeded at densities of 3500, 4000, and 3500 cells/well into a 96-well plate, respectively. The following day, cells were treated with the selected compounds at different concentrations using serial dilutions. Cell viability in 2D assays was evaluated after 72 h by colorimetric MTT (PC3 and HPrEC) or PrestoBlue (LNCaP) assay. Briefly, 0.5 mg/mL of thiazolyl blue tetrazolium bromide (MTT) (Sigma-Aldrich, St. Louis, MO, USA) dissolved in culture medium was added to the wells, and cells were incubated at 37 °C for 2 h. Medium containing unconverted MTT was removed, and 100 μL of MTT solvent (4 mM HCl, 0.1% NP40, in isopropyl alcohol) were added to the wells to solubilize MTT crystals. After 15 min of incubation at r.t., absorbance values at 570 nm were recorded using a Multiskan FC Microplate Photometer (Thermo Fisher Scientific, MA, USA). PrestoBlue reagent (#A13261, Thermo Fisher Scientific, MA, USA) was added to the medium (1:10 v/v) and incubated for 1 h at 37 °C. Absorbance values were determined at 570 and 620 nm. For each cell line, the concentration required to inhibit cell growth by 50% (GI_50_) was determined by using a calibration curve generated from untreated cells at known densities. Three independent experiments were conducted.

#### Cell Cycle Analyses in 2D In Vitro Cultures

Cell cycle analyses were performed on PC3 and LNCaP cell lines. Cells were seeded in a 24-well plate at a density of 37,000 cells/well and 43,000 cells/well for PC3 and LNCaP cells, respectively. After 72 h of incubation, cell-cycle analyses were performed as follows. Propidium iodide-based assay: cells were detached with 1X Trypsin at 37 °C and suspended in 0.5 mL of propidium iodide solution (50 μg/mL propidium iodide, 3.4 mM sodium citrate, 0.1% Triton X-100 in PBS). Afterward, cells were analyzed by Attune NxT Flow Cytometer. *BrdU assay:* cells were incubated with 10 μM BrdU for 1 h at 37 °C and then detached using 1X Trypsin. After washing with PBS1X/Tween-20 0.5% (v/v), cells were incubated with 2 N HCl for 30 min at r.t. and then with 0.1 M borate buffer (pH 8.5) for 5 min at r.t. Cells were washed and then incubated for 1 h at 4 °C with Anti-BrdU primary antibody (BD Bioscience, #347580) at a 1:200 dilution in PBS1X/Tween-20. After washing, cells were incubated with secondary antibody (Alexa Fluor 488 donkey antimouse, Life Technologies, #A21202) diluted 1:75 in TBS/Tween for 1 h in the dark. Finally, cells were resuspended in PI solution (25 μg/mL propidium iodide, 3.4 mM sodium citrate, 9.65 mM NaCl, 0.03% v/v NP40, 100 μg/mL RNase). After 15 min of incubation, cells were analyzed by flow cytometry by an Attune NxT Flow Cytometer.

#### RNA Extraction and RT-qPCR Analysis

RNA was extracted by using TRIzol Reagent (#15596026, Invitrogen), according to the manufacturer’s protocol. For cDNA synthesis, 500 ng of RNA was retrotranscribed with the PrimeScript RT Reagent Kit (#RR037A, Takara Bio). Quantitative RealTime PCRs were performed with SsoAdvanced Universal SYBR Green Supermix (#1725274, Bio-Rad, USA) using the Biorad CFX connect Real-Time PCR Detection System. Oligonucleotide sequences are listed in Table S7. Data were analyzed using the Bio-Rad CFX Maestro 2.0 software (Bio-Rad). mRNA expression levels were normalized to Rps20, and relative fold changes of transcripts were calculated with the formula 2–(ΔΔCt), with untreated cells (Ctr) used as the reference sample.

#### Protein Extraction and Immunoblotting

Whole-cell protein extracts were prepared by lysing cultured cells in 1X SDS sample buffer (25 mM Tris–HCl, pH 6.8, 1.5 mM EDTA, 20% glycerol, 2% SDS, 5% β-mercaptoethanol, 0.0025% Bromophenol blue). Equivalent amounts of cellular extracts were resolved by SDS-PAGE, transferred to nitrocellulose membranes (GE Healthcare) using the Trans-Blot Turbo Transfer System (Bio-Rad, USA), and immunoblotted with the following primary antibodies, diluted 1:1000 in 1X TBS with 1 mg/mL BSA: anti-phospho-Rb (#8516, Cell Signaling), anti-H3 (#PA5-16183, Invitrogen), anti-γH2Ax (#05-638, Millipore), anti-p57 (#PA1876, Boster). Membranes were blotted and scanned with the Amersham Imager AI680 RGB (GE Healthcare) using chemiluminescent detection reagents Westar ηC and Supernova HRP substrates (Cyanagen).

#### 2D Drug Combination Assays

PC3 and HPrEC cells were seeded in 96-well plate at a density of 3500 cells/well. The following day, cells were treated with docetaxel starting from a 30 nM concentration, followed by 3-fold serial dilutions down to 0.014 nM. Treatments were performed either as a single agent or in combination with chlormidazole, which was used at a fixed concentration corresponding to its GI_30_ value in PC3 cells (1.4 μM). After 72 h, cell viability was determined using the MTT assay described above. Dose–response curves were generated and analyzed by nonlinear regression using GraphPad software. For each cell line, GI_50_ values were determined in both conditions tested (docetaxel alone or in combination with chlormidazole) by using a calibration curve generated from untreated cells at known densities. Three independent experiments were conducted.

#### 3D Drug Combination Assay on MTSs

PC3-based MTSs were obtained as described above. Spheroids were treated with docetaxel (0.8 nM, 1.3 nM, 2 nM) or chlormidazole (1.4 μM, 2.2 μM, 4.1 μM), as well as with a combination of 1.3 nM docetaxel and 1.4 μM chlormidazole, corresponding to their GI_30_ values. Untreated spheroids were used as controls (Ctr). The MTSs were maintained for 10 days at 37 °C and 5% CO_2_ in a humidified incubator. Every 3 days, each well was refilled with fresh solutions of the drugs in cell medium or cell medium only (Ctr). Images of the MTSs were captured from day 0 (immediately post-treatment) to day 10. The acquired images were analyzed with ImageJ software to calculate the total spheroid area, and the relative spheroid area, normalized to day 0, was determined as described above.

## Supplementary Material



















## Notes

Supplementary figures S1–S5 and tables S1–S6 report additional computational details; Supplementary figures S6–S7 and table S7 report additional biological details (PDF)
LB similarities in the PC3, DU145 and LNCaP ext-validation data sets (XLSX)
ML classifier predictions in the PC3, DU145 and LNCaP ext-validation data sets (XLSX)
LB similarities, ML classifier predictions, and Combined approach in the PC3, DU145 and LNCaP ext-validation data sets (XLSX)
PC3 data set of compounds from ChEMBL29 (CSV)
DU145 data set of compounds from ChEMBL29 (CSV)
LNCaP data set of compounds from ChEMBL29 (CSV)
PC3, DU145 and LNCaP ext-data sets (XLSX)
DrugBank compounds investigated in this study (XLSX)

## Abbreviation

ADT: androgen deprivation therapy
ARPIs: androgen receptor pathway inhibitors
ROC-AUC: area under the receiver operating characteristic curve
Ctr: control
CRPC: castrate-resistant prostate cancer
CV: cross-validation
ET: Extra Tree
FN: false negative
FP: false positives
HPrEC: prostate epithelial cells
LB: ligand-based
LSD: least significant difference
ML: machine learning
MCC: Matthews correlation coefficient
mCRPC: metastatic castrate-resistant prostate cancer
MTSs: multicellular tumor spheroids
PCa: prostate cancer
SB: structure-based
TTD: therapeutic target database
TN: true negatives
TP: true positives
QSARs: quantitative structure–activity relationships
RFECV: recursive feature elimination CV
RT: room temperature
ULA: ultra-low attachment.

## References

[1] Liu X., Jiang H. (2025). The Global, Regional, and National Prostate Cancer Burden and Trends
from 1990 to 2021, Results from the Global Burden of Disease Study
2021. Front Public Health.

[2] Lancia A., Oderda M., Camilli F., Festa E., Bottero M., Alì E., La Mattina S., Bonzano E., Saddi J., Detti B., Santos Hernandez D. A., Ingrosso G. (2025). Recent Advances in
Androgen Receptor Pathway Inhibitors for Castration-Sensitive Prostate
Cancer. Pharmaceuticals.

[3] Raychaudhuri R., Lin D. W., Montgomery R. B. (2025). Prostate
Cancer: A Review. JAMA.

[4] Kyriakopoulos C. E., Chen Y.-H., Carducci M. A., Liu G., Jarrard D. F., Hahn N. M., Shevrin D. H., Dreicer R., Hussain M., Eisenberger M., Kohli M., Plimack E. R., Vogelzang N. J., Picus J., Cooney M. M., Garcia J. A., Di Paola R. S., Sweeney C. J. (2018). Chemohormonal Therapy in Metastatic
Hormone-Sensitive
Prostate Cancer: Long-Term Survival Analysis of the Randomized Phase
III E3805 CHAARTED Trial. J. Clin. Oncol..

[5] Sayegh N., Swami U., Agarwal N. (2022). Recent Advances in the Management
of Metastatic Prostate Cancer. JCO Oncol. Pract.

[6] Novac N. (2013). Challenges
and Opportunities of Drug Repositioning. Trends
Pharmacol. Sci..

[7] Pinzi L., Bisi N., Rastelli G. (2024). How Drug Repurposing
Can Advance
Drug Discovery: Challenges and Opportunities. Front. Drug Discovery.

[8] Wishart D. S., Feunang Y. D., Guo A. C., Lo E. J., Marcu A., Grant J. R., Sajed T., Johnson D., Li C., Sayeeda Z., Assempour N., Iynkkaran I., Liu Y., Maciejewski A., Gale N., Wilson A., Chin L., Cummings R., Le D., Pon A., Knox C., Wilson M. (2018). DrugBank 5.0: A Major Update to the DrugBank Database
for 2018. Nucleic Acids Res..

[9] Mendez D., Gaulton A., Bento A. P., Chambers J., De Veij M., Félix E., Magariños M. P., Mosquera J. F., Mutowo P., Nowotka M., Gordillo-Marañón M., Hunter F., Junco L., Mugumbate G., Rodriguez-Lopez M., Atkinson F., Bosc N., Radoux C. J., Segura-Cabrera A., Hersey A., Leach A. R. (2019). ChEMBL:
Towards
Direct Deposition of Bioassay Data. Nucleic
Acids Res..

[10] March-Vila E., Pinzi L., Sturm N., Tinivella A., Engkvist O., Chen H., Rastelli G. (2017). On the Integration
of In Silico Drug Design Methods for Drug Repurposing. Front. Pharmacol..

[11] Moraca F., Marzano S., D’Amico F., Lupia A., Fonzo S. D., Vertecchi E., Salvati E., Porzio A. D., Catalanotti B., Randazzo A., Pagano B., Amato J. (2022). Ligand-Based Drug Repurposing
Strategy Identified SARS-CoV-2 RNA G-Quadruplex Binders. Chem. Commun..

[12] Avram, S. ; Curpan, R. F. ; Oprea, T. I. Chapter 7: Cheminformatics Data Mining and Modeling for Drug Repurposing. InDrug Repurposing. Cavalla, D. ; Royal Society of Chemistry, pp. 129–146.2022

[13] Pinzi L., Lherbet C., Baltas M., Pellati F., Rastelli G. (2019). In Silico
Repositioning of Cannabigerol as a Novel Inhibitor of the Enoyl Acyl
Carrier Protein (ACP) Reductase (InhA). Molecules.

[14] Sadybekov A. V., Katritch V. (2023). Computational Approaches
Streamlining Drug Discovery. Nature.

[15] Xu W. (2024). Current Status
of Computational Approaches for Small Molecule Drug Discovery. J. Med. Chem..

[16] Ferreira F. J. N., Carneiro A. S. (2025). AI-Driven Drug Discovery:
A Comprehensive Review. ACS Omega.

[17] Shah, A. ; Jain, M. Limitations and Future Challenges of Computer-Aided Drug Design Methods. In Computer Aided Drug Design (CADD): from Ligand-Based Methods to Structure-Based Approaches; Elsevier, 2022; pp. 283–297. 10.1016/B978-0-323-90608-1.00006-X.

[18] Wassermann A. M., Wawer M., Bajorath J. (2010). Activity Landscape
Representations
for Structure–Activity Relationship Analysis. J. Med. Chem..

[19] Eckert H., Bajorath J. (2007). Molecular Similarity
Analysis in Virtual Screening:
Foundations, Limitations and Novel Approaches. Drug Discovery Today.

[20] Mellor C. L., Marchese Robinson R. L., Benigni R., Ebbrell D., Enoch S. J., Firman J. W., Madden J. C., Pawar G., Yang C., Cronin M. T. D. (2019). Molecular
Fingerprint-Derived Similarity Measures for
Toxicological Read-across: Recommendations for Optimal Use. Regul. Toxicol. Pharmacol..

[21] Sciabola S., Torella R., Nagata A., Boehm M. (2022). Critical Assessment
of State-of-the-Art Ligand-Based Virtual Screening Methods. Mol. Inform..

[22] Fan N., Hirte S., Kirchmair J. (2022). Maximizing the Performance of Similarity-Based
Virtual Screening Methods by Generating Synergy from the Integration
of 2D and 3D Approaches. Int. J. Mol. Sci..

[23] Obaido G., Mienye I. D., Egbelowo O. F., Emmanuel I. D., Ogunleye A., Ogbuokiri B., Mienye P., Aruleba K. (2024). Supervised Machine
Learning in Drug Discovery and Development: Algorithms, Applications,
Challenges, and Prospects. Mach. Learn. Appl..

[24] Svetnik V., Liaw A., Tong C., Culberson J. C., Sheridan R. P., Feuston B. P. (2003). Random Forest: A
Classification and
Regression Tool for Compound Classification and QSAR Modeling. J. Chem. Inf. Comput. Sci..

[25] Bonanni D., Pinzi L., Rastelli G. (2022). Development
of Machine Learning Classifiers
to Predict Compound Activity on Prostate Cancer Cell Lines. J. Cheminf..

[26] Rodrigues T. (2019). The Good,
the Bad, and the Ugly in Chemical and Biological Data for Machine
Learning. Drug Discovery Today: technol..

[27] Klingspohn W., Mathea M., Ter Laak A., Heinrich N., Baumann K. (2017). Efficiency
of Different Measures for Defining the Applicability Domain of Classification
Models. J. Cheminf..

[28] von
Korff M., Sander T. (2022). Limits of Prediction for Machine
Learning in Drug Discovery. Front. Pharmacol..

[29] Cortés-Ciriano I., Firth N. C., Bender A., Watson O. (2018). Discovering Highly
Potent Molecules from an Initial Set of Inactives Using Iterative
Screening. J. Chem. Inf. Model..

[30] Mathai N., Kirchmair J. (2020). Similarity-Based Methods and Machine Learning Approaches
for Target Prediction in Early Drug Discovery: Performance and Scope. Int. J. Mol. Sci..

[31] Ripphausen P., Nisius B., Bajorath J. (2011). State-of-the-art in ligand-based
virtual screening. Drug Discovery Today.

[32] Melville J. L., Burke E. K., Hirst J. D. (2009). Machine learning
in virtual screening. Comb. Chem. High Throughput
Screen.

[33] Bernal L., Pinzi L., Rastelli G. (2023). Identification of Promising
Drug
Candidates against Prostate Cancer through Computationally-Driven
Drug Repurposing. Int. J. Mol. Sci..

[34] Bernal L., Rastelli G., Pinzi L. (2025). Improving
Machine Learning Classification
Predictions through SHAP and Features Analysis Interpretation. J. Chem. Inf. Model..

[35] Lenselink E. B., Ten Dijke N., Bongers B., Papadatos G., van Vlijmen H. W. T., Kowalczyk W., Ijzerman A. P., van Westen G. J. P. (2017). Beyond
the hype: Deep neural networks outperform established methods using
a ChEMBL bioactivity benchmark set. J. Cheminform..

[36] RDKit: Open-Source Cheminformatics; http://www.rdkit.org. (Accessed 22 February 2025).

[37] Pedregosa F., Varoquaux G., Gramfort A., Michel V., Thirion B., Grisel O., Blondel M., Prettenhofer P., Weiss R., Dubourg V., Dubourg V., Vanderplas J., Passos A., Cournapeau D., Brucher M., Perrot M., Duchesnay É (2011). Scikit-Learn:
Machine Learning in Python. J. Mach. Learn.
Res..

[38] Awad M., Fraihat S. (2023). Recursive Feature Elimination with Cross-Validation
with Decision Tree: Feature Selection Method for Machine Learning-Based
Intrusion Detection Systems. J. Sens. Actuator
Netw.

[39] Chicco D., Jurman G. (2020). The Advantages of the
Matthews Correlation Coefficient
(MCC) over F1 Score and Accuracy in Binary Classification Evaluation. BMC Genomics.

[40] Geurts P., Ernst D., Wehenkel L. (2006). Extremely Randomized
Trees. Mach. Learn.

[41] Singh S., Gupta H., Sharma P., Sahi S. (2024). Advances in Artificial
Intelligence (AI)-Assisted Approaches in Drug Screening. Artif. Intell. Chem..

[42] Jasial S., Hu Y., Vogt M., Bajorath J. (2016). Activity-Relevant
Similarity Values
for Fingerprints and Implications for Similarity Searching. F1000research.

[43] Pinzi L., Rastelli G. (2020). Identification of Target
Associations for Polypharmacology
from Analysis of Crystallographic Ligands of the Protein Data Bank. J. Chem. Inf. Model..

[44] McInnes, L. ; Healy, J. ; Melville, J. UMAP: Uniform manifold approximation and projection for dimension reduction arXiv 10.48550/arXiv.1802.03426 2018

[45] Kim K. S., Zhang L., Schmidt R., Cai Z.-W., Wei D., Williams D. K., Lombardo L. J., Trainor G. L., Xie D., Zhang Y., Lippy J., Jeyaseelan R., Wautlet B., Henley B., Gullo-Brown J., Manne V., Hunt J. T., Fargnoli J., Borzilleri R. M. (2008). Discovery
of Pyrrolopyridine–Pyridone Based Inhibitors of Met Kinase:
Synthesis, X-Ray Crystallographic Analysis, and Biological Activities. J. Med. Chem..

[46] Zhang X., Jia D., Liu H., Zhu N., Zhang W., Feng J., Yin J., Hao B., Cui D., Deng Y., Xie D., He L., Li B. (2013). Identification of 5-Iodotubercidin as a Genotoxic Drug
with Anti-Cancer Potential. PLoS One.

[47] Holcakova J., Tomasec P., Bugert J. J., Wang E. C., Wilkinson G. W., Hrstka R., Krystof V., Strnad M., Vojtesek B. (2010). The Inhibitor
of Cyclin-Dependent Kinases, Olomoucine II, Exhibits Potent Antiviral
Properties. Antiviral Chem. Chemother..

[48] Krystof V., Lenobel R., Havlícek L., Kuzma M., Strnad M. (2002). Synthesis
and Biological Activity of Olomoucine II. Bioorg.
Med. Chem. Lett..

[49] Hart S. M., Basu C. (2009). Optimization of a Digoxigenin-Based
Immunoassay System for Gene Detection
in Arabidopsis Thaliana. J. Biomol. Technol.
JBT.

[50] Fromtling R. A. (1988). Overview
of Medically Important Antifungal Azole Derivatives. Clin. Microbiol. Rev..

[51] Richardson, M. D. ; Warnock, D. W. Fungal Infection: Diagnosis and Management; Wiley, 2012. DOI: 10.1002/9781118321492.

[52] Musiol R., Kowalczyk W. (2012). Azole Antimycotics
- A Highway to New Drugs or a Dead
End?. Curr. Med. Chem..

[53] Shafiei M., Peyton L., Hashemzadeh M., Foroumadi A. (2020). History of
the Development of Antifungal Azoles: A Review on Structures, SAR,
and Mechanism of Action. Bioorg. Chem..

[54] Sheehan D. J., Hitchcock C. A., Sibley C. M. (1999). Current and Emerging Azole Antifungal
Agents. Clin. Microbiol. Rev..

[55] Odds F. (2003). Antifungal
Agents: Their Diversity and Increasing Sophistication. Mycologist.

[56] Satija G., Sharma B., Madan A., Iqubal A., Shaquiquzzaman M., Akhter M., Parvez S., Khan M. A., Alam M. M. (2022). Benzimidazole
Based Derivatives as Anticancer Agents: Structure Activity Relationship
Analysis for Various Targets. J. Heterocycl.
Chem..

[57] Gaba M., Mohan C. (2016). Development of drugs based on imidazole and benzimidazole bioactive
heterocycles: Recent advances and future directions. Med. Chem. Res..

[58] Phan N.-K.-N., Huynh T.-K.-C., Nguyen H.-P., Le Q.-T., Nguyen T.-C.-T., Ngo K.-K.-H., Nguyen T.-H.-A., Ton K. A., Thai K.-M., Hoang T.-K.-D. (2023). Exploration of Remarkably Potential
Multitarget-Directed
N-Alkylated-2-(Substituted Phenyl)-1H-Benzimidazole Derivatives as
Antiproliferative, Antifungal, and Antibacterial Agents. ACS Omega.

[59] Weng N., Zhang Z., Tan Y., Zhang X., Wei X., Zhu Q. (2023). Repurposing Antifungal
Drugs for Cancer Therapy. J. Adv. Res..

[60] Figg W. D., Woo S., Zhu W., Chen X., Ajiboye A. S., Steinberg S. M., Price D. K., Wright J. J., Parnes H. L., Arlen P. M., Gulley J. L., Dahut W. L. (2010). A Phase I Clinical Study of High
Dose Ketoconazole Plus Weekly Docetaxel for Metastatic Castration
Resistant Prostate Cancer. J. Urol..

[61] Womble P. R., Van Veldhuizen P. J., Nisbet A. A., Reed G. A., Thrasher J. B., Holzbeierlein J. M. (2011). A Phase II Clinical Trial of Neoadjuvant Ketoconazole
and Docetaxel Chemotherapy Before Radical Prostatectomy in High Risk
Patients. J. Urol..

[62] Rehman O. U., Nadeem Z. A., Fatima E., Akram U., Imran H., Husnain A., Nadeem A., Rasheed W. (2024). The Efficacy of Ketoconazole
Containing Regimens in Castration-Resistant Prostate Cancer: A Systematic
Review and Meta-Analysis. Clin Genitourin Cancer.

[63] Patel V., Liaw B., Oh W. (2018). The Role of
Ketoconazole in Current
Prostate Cancer Care. Nat. Rev. Urol..

[64] Ding V. A., Zhu Z., Xiao H., Wakefield M. R., Bai Q., Fang Y. (2016). The Role of
IL-37 in Cancer. Med. Oncol..

[65] Martini A., Parikh A. B., Sfakianos J. P., Montorsi F., Galsky M. D., Oh W. K., Tsao C.-K. (2021). Predicting
Toxicity-Related Docetaxel
Discontinuation and Overall Survival in Metastatic Castration-Resistant
Prostate Cancer: A Pooled Analysis of Open Phase 3 Clinical Trial
Data. Prostate Cancer Prostatic Dis..

[66] Wei Q., Dunbrack R. L. (2013). The Role
of Balanced Training and Testing Data Sets
for Binary Classifiers in Bioinformatics. PLoS
One.

