# Supplementary material for: A Combined Chemoinformatics- and Machine Learning-Based Approach Identifies Chlormidazole as a Drug Repurposing Candidate against Aggressive Prostate Cancer
Source: J Med Chem. 2026 Aug 4;69(15):17944–60. doi: 10.1021/acs.jmedchem.5c03833 (PMC13492279; doi:10.1021/acs.jmedchem.5c03833)
Supplement: Supplementary file 1 [file jm5c03833_si_001.pdf]

SUPPORTING INFORMATION

A Combined Chemoinformatics– and Machine Learning–Based Approach Identifies Chlormidazole as a Drug Repurposing Candidate Against Aggressive Prostate Cancer

Leonardo Bernal,[Δ1,2] *Luca Pinzi,*[Δ1*] *Tommaso Martinelli,*[1] *Arianna Rinaldi,*[1] *Isabella Piccinini,*[1] *Silvia Belluti,*[1] *Nicolò Bisi,*[1] *Carol Imbriano,*[1] *Giulio Rastelli*[1]*

[1] Department of Life Sciences, University of Modena and Reggio Emilia, Via Giuseppe Campi 103, 41125 Modena, Italy.

[2] Clinical and Experimental Medicine PhD Program, University of Modena and Reggio Emilia, Via Giuseppe Campi 287, 41125 Modena, Italy.

[Δ] Equally contributing authors

Table of Contents

Tables

Table S1: Hyperparameters optimized during the development of the machine learning models S4
Table S2: RDKit molecular descriptors selected by RFECV S5
Table S3: Performance metrics obtained on their respective test sets for the ML models reported in L. Bernal et al., *J. Chem. Inf. Model.* 2025, 65, 11716−11732 S6
Table S4: Precision values obtained by the similarity screenings, the ML classifiers, and the *Combined approach* on the *ext-validation* set for each PCa cell line S7
Table S5: Relative percentages of True Positive, False Positive, True Negative and False Negative predictions obtained by the ML models and the *Combined approach* in the PC3, DU145, and LNCaP datasets S8
Table S6: DrugBank compounds predicted to be active across all PCa cell lines according to our *Combined approach* S9
Table S7: Sequences of the oligonucleotides used in the RT-qPCR experiments S10

Figures

Figure S1: Classification of compounds as "active" or "inactive" according to a 10 μM threshold S11
Figure S2: Venn diagram illustrating the number of compounds with reported antiproliferative activity (ChEMBL29) in the PC3, DU145, and LNCaP datasets, and their intersections S12
Figure S3: Distribution of the compounds in the training and test sets according to MACCS and ECFP4 fingerprint-based similarity scores in the PC3, DU145 and LNCaP datasets S13
Figure S4: Performance metrics of the machine learning classifiers for the PC3, DU145 and LNCaP cell lines S14
Figure S5: Applicability domain analyses of the PC3, DU145, and LNCaP training/test and *ext-validation* datasets S15
Figure S6: RT-qPCR analysis of the indicated transcripts in PC3 cells after 72 h of treatment with chlormidazole at its GI_{50} dose S16
Figure S7: Dose-dependent curves of ketoconazole (*panel A)* and its corresponding GI_{50} values (*panel B*) in PC3, LNCaP, and HPrEC cells after 72 h of treatment S17

Table S1. Hyperparameters optimized during the development of the machine learning models. The table reports the hyperparameters, the values explored, and the optimal values selected for each cell lines.

Hyperparameter	***Values explored***	***PC3***		***DU145***		***LNCAP***	
		**1st tuning:**	**2nd tuning:**	**1st tuning:**	**2nd tuning:**	**1st tuning:**	**2nd tuning:**
Number of estimators	100; 150; 200; 250; 300; 350; 400; 450; 500	400	500	300	400	150	150
Max depth	5; 10; 15	15	15	15	15	10	15
Min samples split	10; 20; 30	10	10	10	10	10	10
Min samples leaf	10; 15; 20	10	10	10	10	10	10
Max features	sqrt; log2; None	None	None	None	None	None	None
Bootstrap	True; False	False	False	False	False	False	False
Criterion	gini; entropy	entropy	entropy	gini	gini	gini	gini
Class weight	None; balanced	balanced	balanced	balanced	balanced	balanced	None

Table S2. RDKit molecular descriptors selected by RFECV.

Dataset	*N° RDKit features retained*	*RDKit features retained*
PC3	**108**	MaxAbsEStateIndex, MaxEStateIndex, MinAbsEStateIndex, MinEStateIndex, qed, SPS, MaxPartialCharge, MinPartialCharge, MaxAbsPartialCharge, MinAbsPartialCharge, FpDensityMorgan1, FpDensityMorgan2, FpDensityMorgan3, AvgIpc, BalabanJ, Chi2n, Chi3v, Chi4n, Chi4v, HallKierAlpha, Kappa2, Kappa3, PEOE_VSA1, PEOE_VSA10, PEOE_VSA11, PEOE_VSA12, PEOE_VSA13, PEOE_VSA14, PEOE_VSA2, PEOE_VSA3, PEOE_VSA6, PEOE_VSA7, PEOE_VSA8, PEOE_VSA9, SMR_VSA10, SMR_VSA3, SMR_VSA4, SMR_VSA5, SMR_VSA6, SMR_VSA7, SMR_VSA9, SlogP_VSA1, SlogP_VSA10, SlogP_VSA11, SlogP_VSA2, SlogP_VSA3, SlogP_VSA4, SlogP_VSA5, SlogP_VSA6, SlogP_VSA8, TPSA, EState_VSA2, EState_VSA8, EState_VSA9, VSA_EState2, VSA_EState3, VSA_EState4, VSA_EState6, VSA_EState8, VSA_EState9, FractionCSP3, HeavyAtomCount, NHOHCount, NOCount, NumAliphaticCarbocycles, NumAliphaticHeterocycles, NumAliphaticRings, NumAromaticCarbocycles, NumAromaticHeterocycles, NumAromaticRings, NumHAcceptors, NumHDonors, NumHeteroatoms, NumRotatableBonds, NumSaturatedHeterocycles, NumSaturatedRings, RingCount, MolLogP, MolMR, fr_Ar_N, fr_C_O, fr_C_O_noCOO, fr_NH0, fr_NH1, fr_N_O, fr_allylic_oxid, fr_amide, fr_aniline, fr_aryl_methyl, fr_benzene, fr_bicyclic, fr_ether, fr_furan, fr_imidazole, fr_ketone, fr_lactone, fr_methoxy, fr_para_hydroxylation, fr_pyridine, fr_quatN, fr_sulfide, fr_urea, 3d_Asphericity, 3d_InertialShapeFactor, 3d_NPR1, 3d_NPR2, 3d_PBF, 3d_SpherocityIndex
LNCaP	**50**	MinEStateIndex, SPS, FpDensityMorgan1, FpDensityMorgan2, AvgIpc, BertzCT, Chi0n, Chi1, Chi1n, Chi2n, Chi3n, Chi4n, LabuteASA, PEOE_VSA11, PEOE_VSA3, SMR_VSA3, SMR_VSA5, SMR_VSA6, SMR_VSA7, SlogP_VSA10, SlogP_VSA4, SlogP_VSA5, SlogP_VSA7, SlogP_VSA8, EState_VSA1, VSA_EState6, VSA_EState7, FractionCSP3, HeavyAtomCount, NHOHCount, NumAliphaticHeterocycles, NumAromaticHeterocycles, NumAromaticRings, NumSaturatedHeterocycles, MolMR, fr_Ar_N, fr_C_O_noCOO, fr_NH0, fr_NH1, fr_NH2, fr_alkyl_halide, fr_allylic_oxid, fr_aryl_methyl, fr_benzene, fr_imidazole, fr_ketone, fr_morpholine, fr_urea, 3d_Asphericity, 3d_InertialShapeFactor
DU145	**196**	MaxAbsEStateIndex, MaxEStateIndex, MinAbsEStateIndex, MinEStateIndex, qed, SPS, MolWt, HeavyAtomMolWt, ExactMolWt, NumValenceElectrons, NumRadicalElectrons, MaxPartialCharge, MinPartialCharge, MaxAbsPartialCharge, MinAbsPartialCharge, FpDensityMorgan1, FpDensityMorgan2, FpDensityMorgan3, AvgIpc, BalabanJ, BertzCT, Chi0, Chi0n, Chi0v, Chi1, Chi1n, Chi1v, Chi2n, Chi2v, Chi3n, Chi3v, Chi4n, Chi4v, HallKierAlpha, Kappa1, Kappa2, Kappa3, LabuteASA, PEOE_VSA1, PEOE_VSA10, PEOE_VSA11, PEOE_VSA12, PEOE_VSA13, PEOE_VSA14, PEOE_VSA2, PEOE_VSA3, PEOE_VSA4, PEOE_VSA5, PEOE_VSA6, PEOE_VSA7, PEOE_VSA8, PEOE_VSA9, SMR_VSA1, SMR_VSA10, SMR_VSA2, SMR_VSA3, SMR_VSA4, SMR_VSA5, SMR_VSA6, SMR_VSA7, SMR_VSA9, SlogP_VSA1, SlogP_VSA10, SlogP_VSA11, SlogP_VSA12, SlogP_VSA2, SlogP_VSA3, SlogP_VSA4, SlogP_VSA5, SlogP_VSA6, SlogP_VSA7, SlogP_VSA8, TPSA, EState_VSA1, EState_VSA10, EState_VSA11, EState_VSA2, EState_VSA3, EState_VSA4, EState_VSA5, EState_VSA6, EState_VSA8, EState_VSA9, VSA_EState1, VSA_EState10, VSA_EState2, VSA_EState3, VSA_EState4, VSA_EState5, VSA_EState6, VSA_EState7, VSA_EState8, VSA_EState9, FractionCSP3, HeavyAtomCount, NHOHCount, NOCount, NumAliphaticCarbocycles, NumAliphaticHeterocycles, NumAliphaticRings, NumAromaticCarbocycles, NumAromaticHeterocycles, NumAromaticRings, NumHAcceptors, NumHDonors, NumHeteroatoms, NumRotatableBonds, NumSaturatedCarbocycles, NumSaturatedHeterocycles, NumSaturatedRings, RingCount, MolLogP, MolMR, fr_Al_COO, fr_Al_OH, fr_Al_OH_noTert, fr_ArN, fr_Ar_COO, fr_Ar_N, fr_Ar_NH, fr_Ar_OH, fr_COO, fr_COO2, fr_C_O, fr_C_O_noCOO, fr_C_S, fr_Imine, fr_NH0, fr_NH1, fr_NH2, fr_N_O, fr_Ndealkylation1, fr_Ndealkylation2, fr_Nhpyrrole, fr_SH, fr_alkyl_carbamate, fr_alkyl_halide, fr_allylic_oxid, fr_amide, fr_amidine, fr_aniline, fr_aryl_methyl, fr_azide, fr_azo, fr_benzene, fr_bicyclic, fr_dihydropyridine, fr_epoxide, fr_ester, fr_ether, fr_furan, fr_guanido, fr_halogen, fr_hdrzine, fr_hdrzone, fr_imidazole, fr_imide, fr_isothiocyan, fr_ketone, fr_ketone_Topliss, fr_lactam, fr_lactone, fr_methoxy, fr_morpholine, fr_nitrile, fr_nitro, fr_nitro_arom, fr_nitroso, fr_oxime, fr_para_hydroxylation, fr_phenol, fr_phenol_noOrthoHbond, fr_piperdine, fr_piperzine, fr_priamide, fr_pyridine, fr_quatN, fr_sulfide, fr_sulfonamd, fr_sulfone, fr_tetrazole, fr_thiazole, fr_thiocyan, fr_thiophene, fr_urea, 3d_Asphericity, 3d_Eccentricity, 3d_InertialShapeFactor, 3d_NPR1, 3d_NPR2, 3d_PBF, 3d_PMI1, 3d_PMI2, 3d_PMI3, 3d_RadiusOfGyration, 3d_SpherocityIndex

Table S3. Performance metrics obtained on their respective test sets for the ML models reported in L. Bernal et al., *J. Chem. Inf. Model.* 2025, 65, 11716−11732.

Cell line	*Algorithm*	*Accuracy*	*MCC*	*Precision*	*Recall*	*ROC AUC*	*F1 Score*
DU-145	ET	0.80	0.60	0.80	0.86	0.88	0.83
	GBM	0.80	0.60	0.81	0.85	0.88	0.83
	RF	0.80	0.59	0.83	0.81	0.87	0.82
	XGB	0.78	0.55	0.78	0.84	0.86	0.81
LNCaP	ET	0.81	0.59	0.88	0.81	0.90	0.85
	GBM	0.84	0.62	0.84	0.92	0.91	0.88
	RF	0.81	0.59	0.88	0.84	0.90	0.86
	XGB	0.83	0.62	0.85	0.91	0.91	0.88
PC3	ET	0.79	0.58	0.86	0.76	0.88	0.81
	GBM	0.80	0.59	0.81	0.87	0.88	0.84
	RF	0.80	0.59	0.85	0.80	0.88	0.82
	XGB	0.78	0.54	0.78	0.86	0.86	0.82

Table S4. Precision values obtained by the similarity screenings, the ML classifiers, and the *Combined approach* on the *ext-validation* set for each PCa cell line.

	PRECISION		
	**PC3**	**DU145**	**LNCaP**
Ligand-based similarity	0.79	0.72	0.48
ML classification	0.70	0.68	0.80
Combined approach	0.82	0.78	0.61

Table S5: Relative percentages of True Positive, False Positive, True Negative and False Negative predictions provided by the ML models and the *Combined approach* in the PC3, DU145 and LNCaP datasets.

Dataset	Class of prediction of the ML classifier	Percentages of Predictions (ML approach)*	Percentages of Predictions (CA)*
PC3	TP	41.7	54.6
	TN	22.9	13.4
	FP	15.7	14.9
	FN	19.7	17.1
DU145	TP	48.3	57.8
	TN	22.0	20.0
	FP	20.5	18.1
	FN	9.2	4.2
LNCaP	TP	57.5	51.4
	TN	16.8	20.2
	FP	14.2	22.0
	FN	11.4	6.4

Note: * relative to the total predictions. TP: True Positive; TN: True Negative; FP: False Positive; FN: False Negative.

Table S6. DrugBank compounds predicted to be active across all three PCa cell lines according to our *Combined approach*. Each entry reports the number of compounds similar to active molecules (< 10 µM threshold) resulting from the LB similarity screenings, along with their primary therapeutic indications. DrugBank ligands selected for biological assays are highlighted in bold.

DRUGBANK ID	Molecule Name	N° of compounds similar to active molecules with PCa antiproliferative effects	Activity
DB13611	**Chlormidazole**	**2**	**Antifungal**
DB13103	Gpx-150	1	Antitumor
DB04604	**5-iodotubercidin**	**18**	**Antitumor**
DB06896	**Bms-2**	**4**	**Antitumor**
DB02427	2,4-diamino-6-[n-(2,5-dimethoxybenzyl)-n-methylamino]quinazoline	3	Antitumor
DB03671	**Digoxigenin**	**3**	**Other uses (diagnostics)**
DB04011	2-(4-dimethylaminophenyl)-5-(4-methyl-1-piperazinyl)-2,5-bi-benzimidazole	3	DNA intercalante
DB03351	Sri-9439	2	Antitumor
DB03987	2,4-diamino-6-[n-(3,5-dimethoxybenzyl)-n-methylamino]pyrido[2,3-d]pyrimidine	2	Antitumor
DB01958	5-[4-tert-butylphenylsulfanyl]-2,4-quinazolinediamine	1	Antibacterial & antifungal
DB02583	N6-(2,5-dimethoxy-benzyl)-n6-methyl-pyrido[2,3-d]pyrimidine-2,4,6-triamine	1	Antitumor
DB04616	Tacrine(8)-4-aminoquinoline	1	Gene signaling modulation
DB04662	**Olomoucine ii**	**1**	**Antitumor & antiviral**
DB07153	6-methyl-5-[3-methyl-3-(3,4,5-trimethoxyphenyl)but-1-yn-1-yl]pyrimidine-2,4-diamine	1	Antitumor
DB07769	S-3-(4-fluorophenoxy)-2-hydroxy-2-methyl-n-[4-nitro-3-(trifluoromethyl)phenyl]propanamide	1	Male contraceptive / Osteoporosis
DB15653	Islatravir	1	Antiviral

Table S7. Sequences of the oligonucleotides used in the RT-qPCR experiments.

Target	Sequence (5'-3')
Cyclin B1	***Fw***: CACTTCCTTCGGAGAGCATC
	Rev: CAGGTGCTGCATAACTGGAA
Cyclin D1	***Fw:*** CCTGTCCTCTACCGCCTCA
	Rev: TCCTCCTCTTCCTCCTCCTC
P16	***Fw:*** GCCCAACGCACCGAATAG
	Rev: ACGGGTCGGGTGAGAGTG
P21	***Fw:*** TGACCCTGAAGTGAGCACAG
	Rev: GGGAAAAGGCTCAACACTGA
P27	***Fw:*** CAGACGGGGTTAGCGGAG
	Rev: GCTCCACAGAACCGGCAT
P53	***Fw:*** CCCAGAAAACCTACCAGGGC
	Rev: GAAGCGCTCACGCCCACG
P57	***Fw:*** ACCTTCCCAGTACTAGTGCG
	Rev: AAGTCGTAATCCCAGCGGTT
BAX	***Fw:*** GTCCGGGGAGCAGCCCAGAG
	Rev: CTCCATGTTACTGTCCAGTTCGTCC
NOXA	***Fw:*** CGGGCTCCAGCAGAGCTGGAAG
	Rev: GATAATCTGGGAAAACTTGAACAG
RPS20	***Fw:*** GCGCCTCTTATCAAAGTCAGC
	Rev: CGGAAAAACACCCGTGGAG

Figure S1. Classification of compounds as "active" or "inactive" according to a 10 μM threshold. *Panel A* shows the percentages of active and inactive compounds (GI_{50} values, 10 μM threshold) in the datasets used for training and testing of the machine learning models. Active and inactive compounds are colored in blue and orange, respectively. *Panel B* shows the distribution of GI_{50} values in the corresponding datasets.

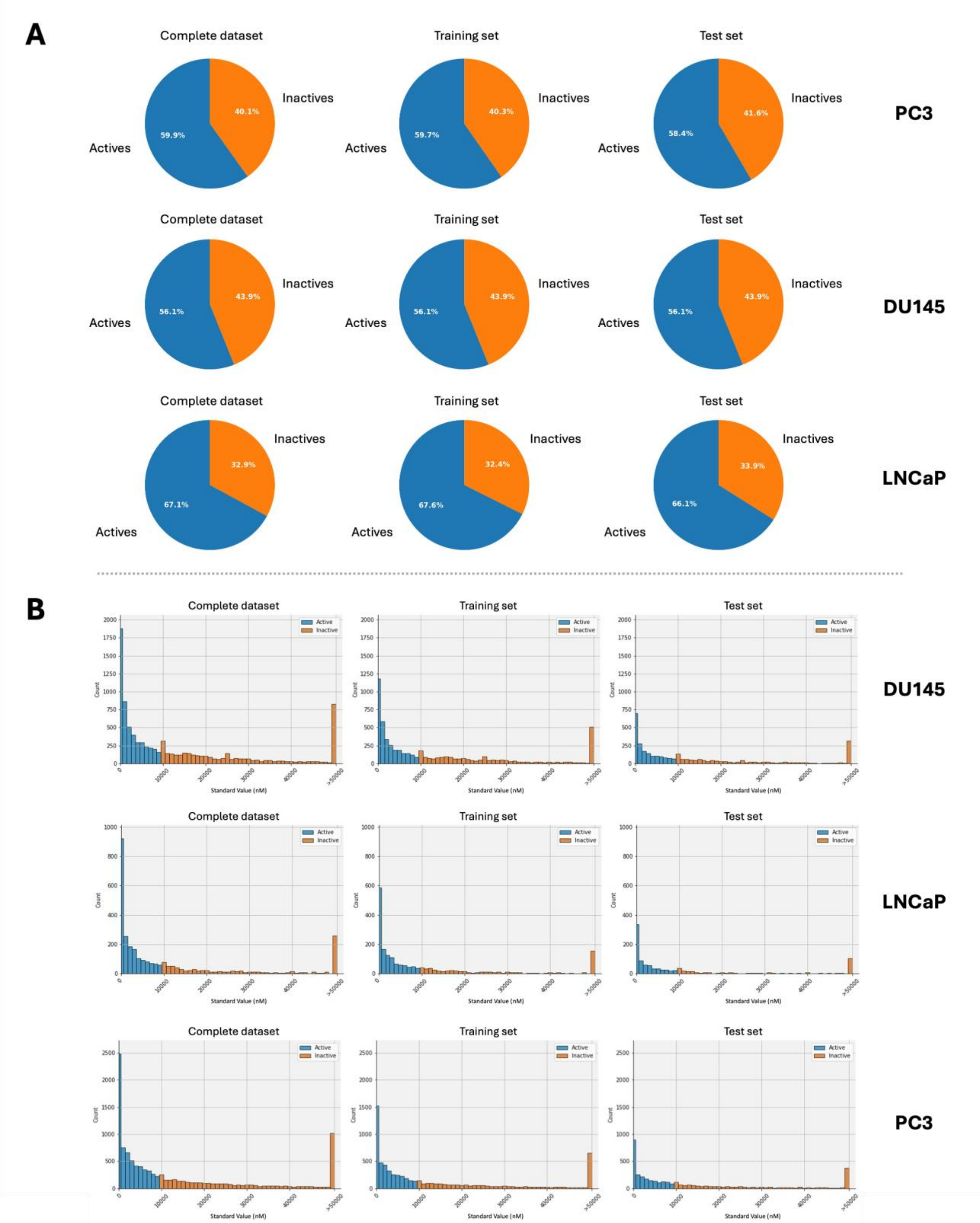

Figure S2. Venn diagram illustrating the number of compounds with reported antiproliferative activity (ChEMBL29) in the PC3, DU145, and LNCaP datasets, and their intersections.

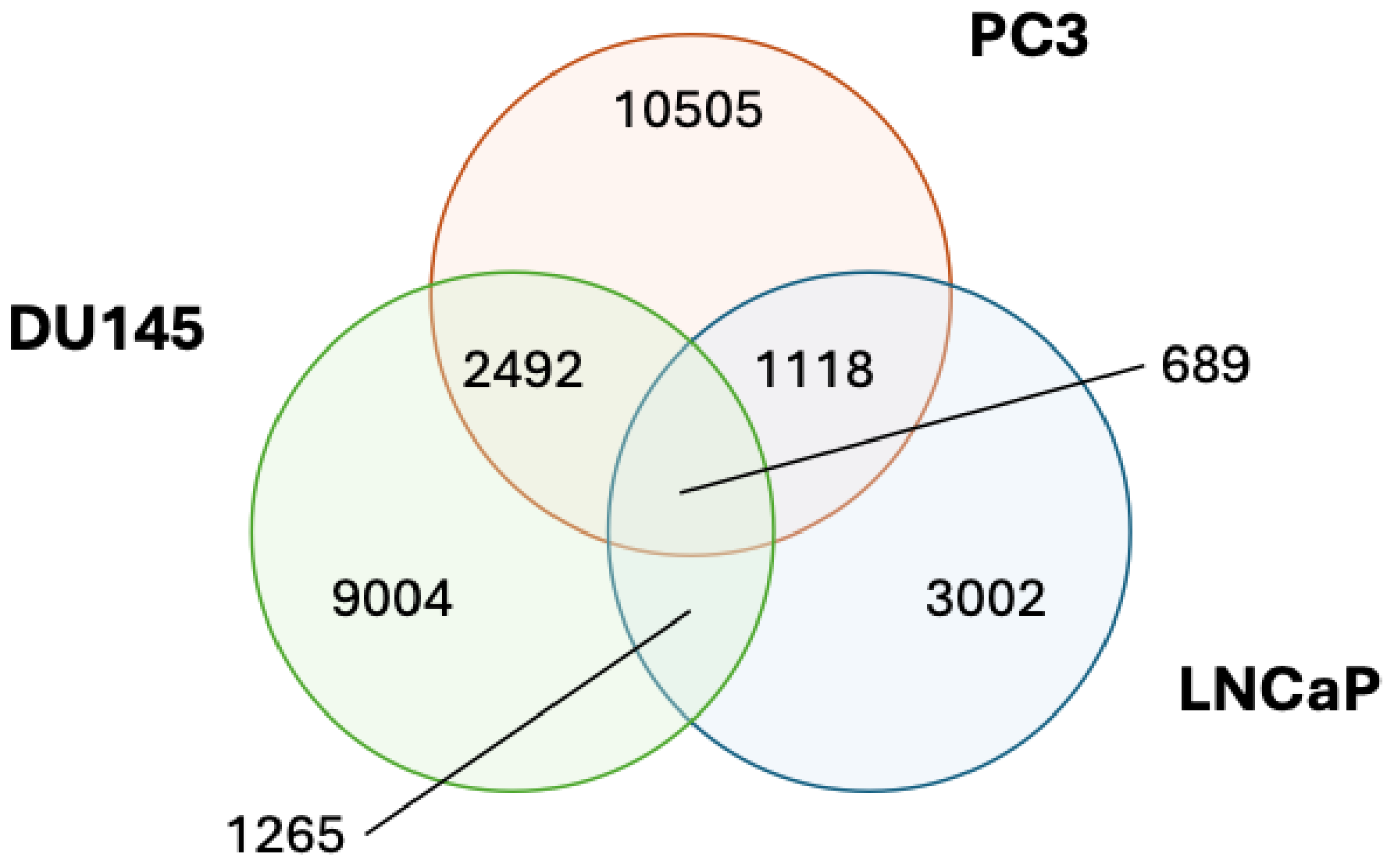

Figure S3. Distribution of the compounds in the training and test sets according to MACCS and ECFP4 fingerprint-based similarity scores in the PC3, DU145, and LNCaP datasets. The graphs highlight a significant overlap between the training and test sets, indicative of a balanced chemical diversity representation.

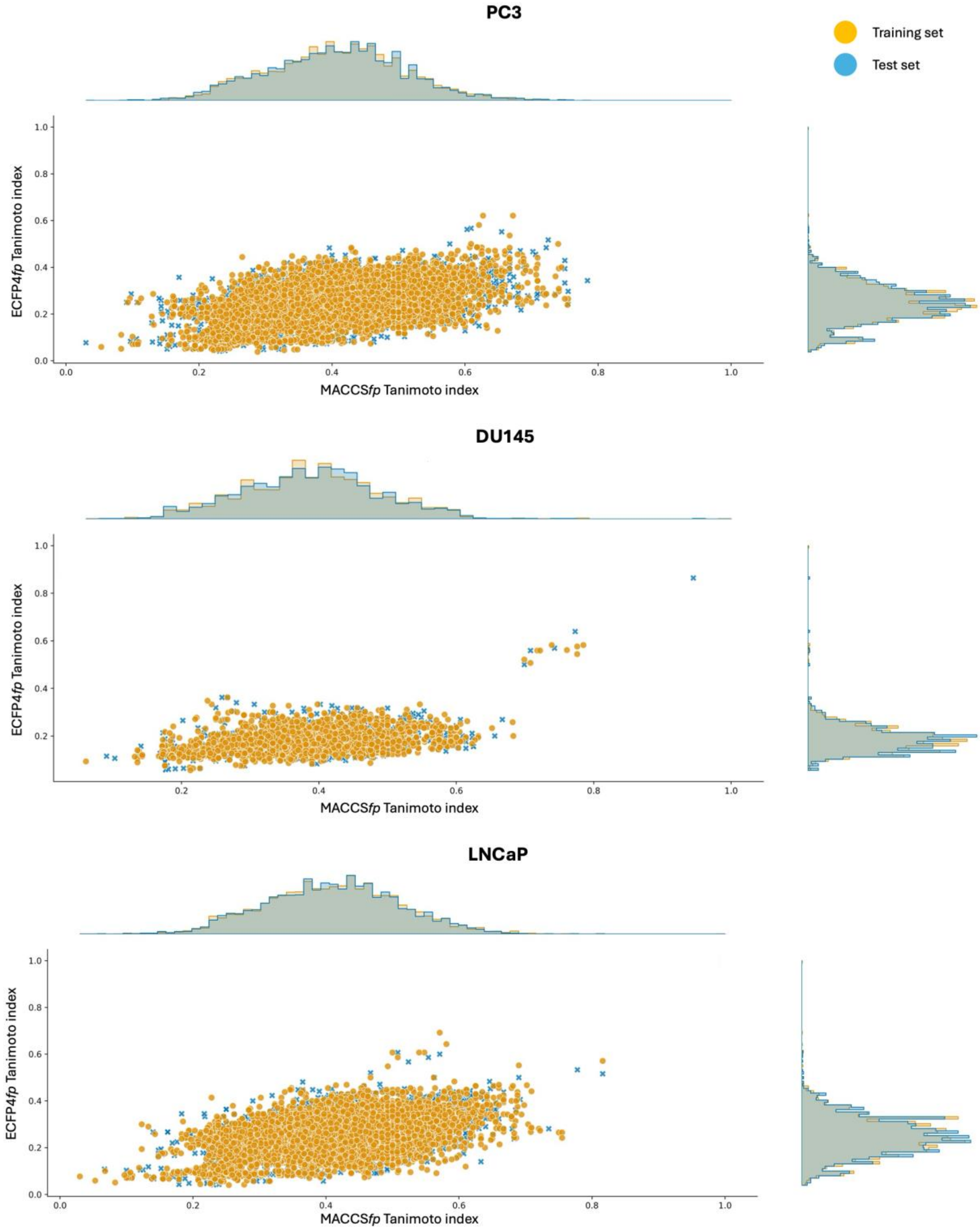

Figure S4. Performance metrics of the machine learning classifiers for the PC3, DU145, and LNCaP cell lines. The histograms show the values of Accuracy, MCC, Precision, Recall, F1-score and ROC AUC in both the training and test sets.

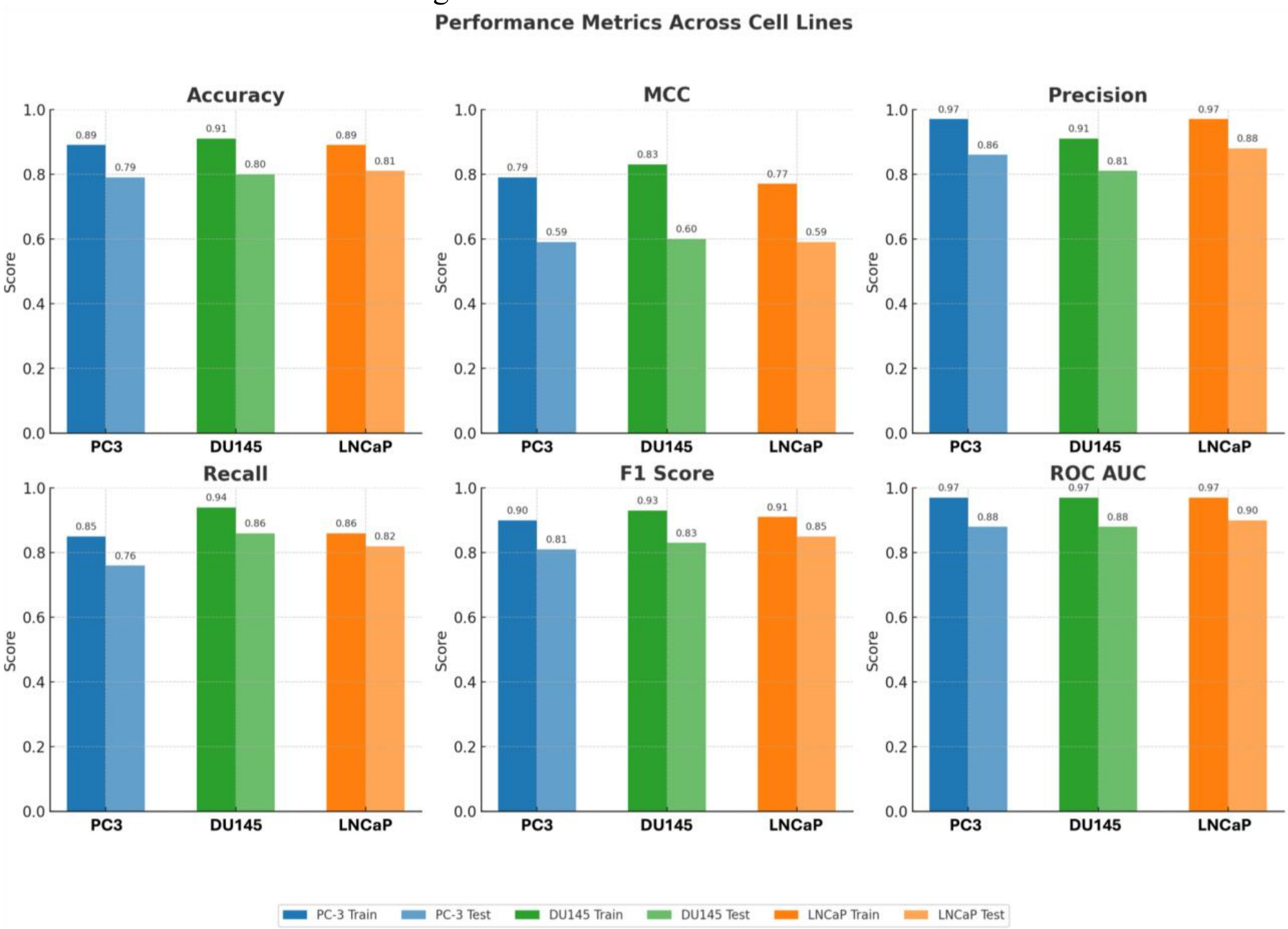

Figure S5. Applicability domain analyses of the PC3, DU145, and LNCaP training/test and *ext-validation* datasets. Compounds in the *ext-validation* dataset showed overall moderate (ECFP4 fingerprints) to low (MACCS fingerprints) degree of similarity with respect to those employed for models' training. Red dashed lines in the "*Ext-validation to training/test sets nearest-neighbor similarity*" plots indicate commonly accepted thresholds of similarity, i.e. MACCS similarity ≥ 0.80 and ECFP4 similarity ≥ 0.30. 3D-UMAP overlay plots report the 3D spatial distribution of the compounds in training/test and *ext-validation* datasets. Compounds in the training/test are shown as grey dots, while *ext-validation* ligands within or outside the applicability domain of the training/test dataset are depicted as blue dots and pink crosses, respectively.

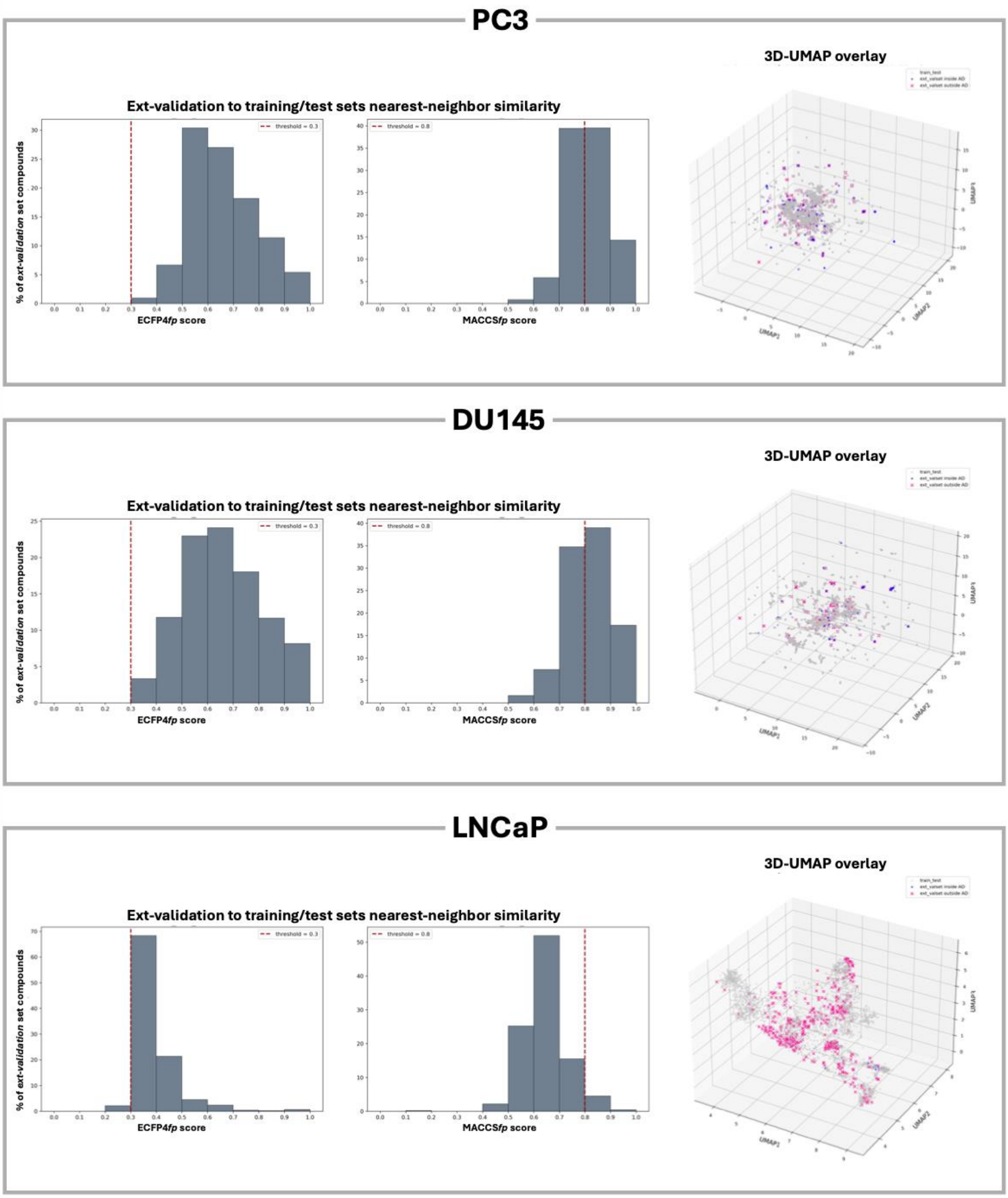

Figure S6. RT-qPCR analysis of the indicated transcripts in PC3 cells after 72 h of treatment with chlormidazole at its GI_{50} dose. *RPS20* was used as reference gene and normalized mRNA levels are reported as fold change vs untreated cells (Ctr), arbitrarily set to 1. Data represent mean ± SEM (Student's t-test, n=3: $*p<0.05$, $***\ p < 0.001$).

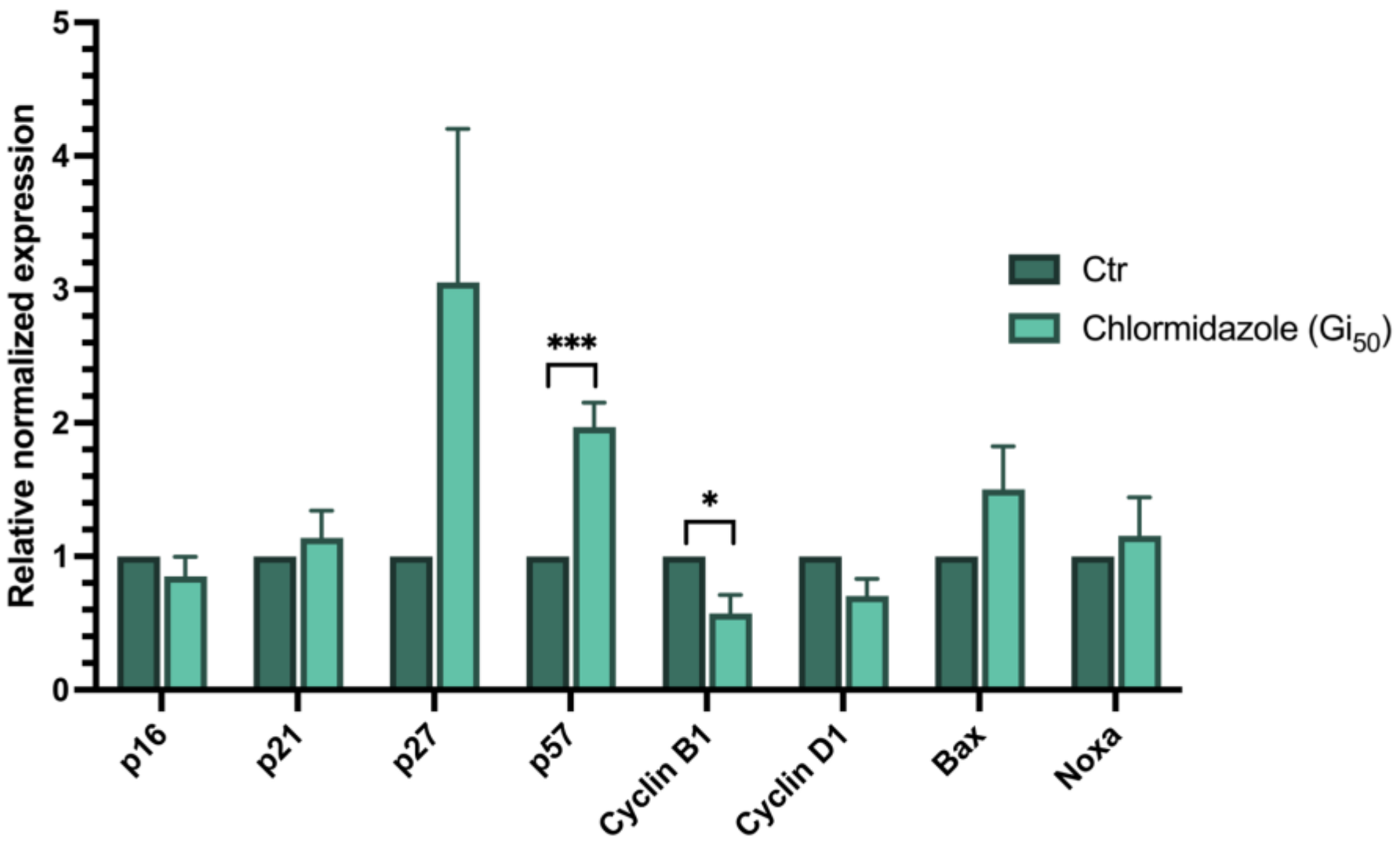

Figure S7. Dose-dependent curves of ketoconazole (*panel A)* and its corresponding GI_{50} values (*panel B*) in PC3, LNCaP and HPrEC cells after 72 h of treatment.

A

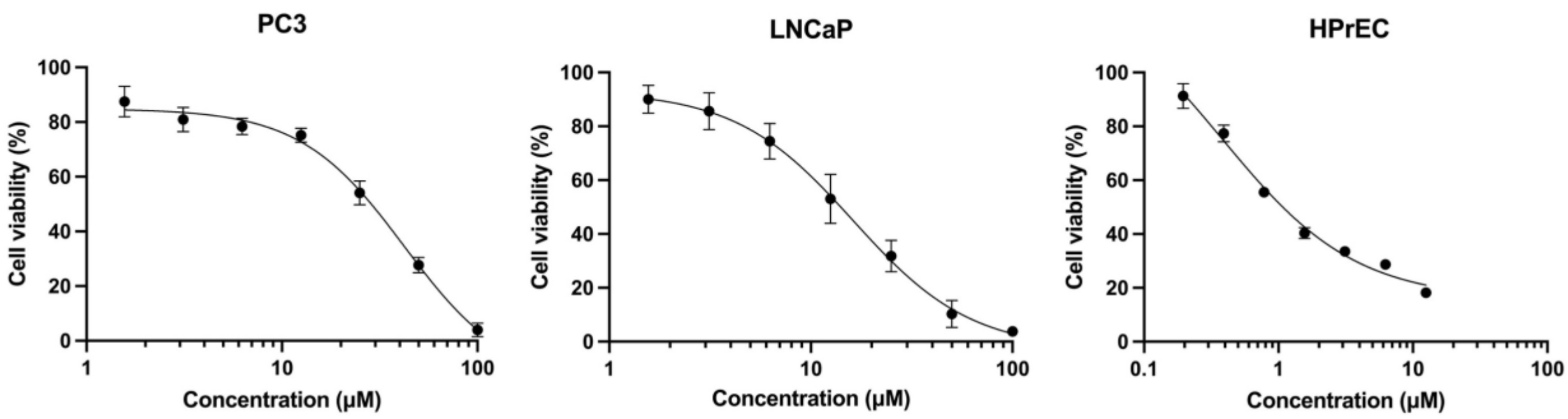

B

Compound Name	2D structure	PC3 GI_{50} (µM)	LNCaP GI_{50} (µM)	HPrEC GI_{50} (µM)	Therapeutic Indication
**Ketoconazole**	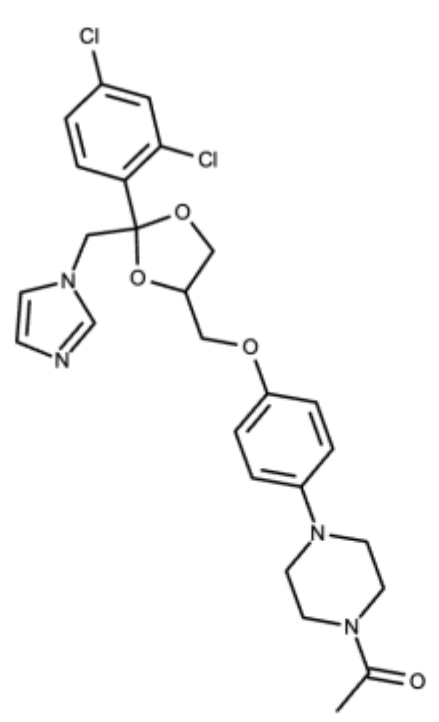	31.8 ± 2	16.1 ± 3.2	1.5 ± 0.3	Antifungal
